# Supplementary material for: Extensive introgression and mosaic genomes of Mediterranean endemic lizards
Source: Nat Commun. 2021 May 12;12:2762. doi: 10.1038/s41467-021-22949-9 (PMC8114931; doi:10.1038/s41467-021-22949-9)
Supplement: Supplementary file 1 — Supplementary Information [file 41467_2021_22949_MOESM1_ESM.pdf]

Supplementary Information

Extensive introgression and mosaic genomes of Mediterranean endemic lizards

Weizhao Yang, Nathalie Feiner, Catarina Pinho, Geoffrey M. While, Antigoni Kaliontzopoulou, D. James Harris, Daniele Salvi and Tobias Uller

Supplementary Figures

Supplementary Fig. 1 Distribution of missing rate of SNVs for each *Podarcis* lineage.
Supplementary Fig. 2 Distribution of genetic diversity (π) for each *Podarcis* lineage.
Supplementary Fig. 3 PCA plot of genetic distance for all *Podarcis* lineages.
Supplementary Fig. 4 Summary of node support across methodologies.
Supplementary Fig. 5 Consistency of topologies among different window sizes.
Supplementary Fig. 6 Discrepancy between nuclear and mitochondrial trees.
Supplementary Fig. 7 Timing of diversifications.
Supplementary Fig. 8 Maximum-likelihood (ML) tree of *Podarcis* lizards based on the concatenating SNVs across the whole genome (WGS dataset).
Supplementary Fig. 9 Maximum-likelihood (ML) tree based on only SNVs from sequences of protein-coding genes (CDS dataset).
Supplementary Fig. 10 Maximum-likelihood tree inferred from the mitochondrial genome (mtDNA dataset).
Supplementary Fig. 11 Overview of admixture across *Podarcis*.
Supplementary Fig. 12 Introgression events revealed by qpGraph and phyloNet.
Supplementary Fig. 13 Pattern of protein-coding gene evolution measured by dN/dS ratio.
Supplementary Fig. 14 Reticulate evolution between *P. muralis* - Iberian clade and Western Islands clade.
Supplementary Fig. 15 Model test for introgression between Western Islands and Sicilian-Maltese groups.
Supplementary Fig. 16 Model test for introgression in *P. tauricus* species complex.
Supplementary Fig. 17 Model test for introgression of the eastern lineage of *P. peloponnesiacus*.
Supplementary Fig. 18 Model test for hybrid origin of *P. guadarramae*.
Supplementary Fig. 19 Model test for reticulate evolution of *P. vaucheri*.
Supplementary Fig. 20 Model test for reticulate evolution of *P. carbonelli*.
Supplementary Fig. 21 Introgression for Spanish lineage of *P. muralis*.
Supplementary Fig. 22 Length distribution of genomic blocks for which *P. muralis* and the Sicilian subclade share the same ancestry.
Supplementary Fig. 23 Timing of diversifications based on the WGS dataset and on mitochondrial DNA.
Supplementary Fig. 24 Historical dynamics in effective population size inferred by PSMC for two lineages of *P. muralis*.

Supplementary Fig. 25 Historical dynamics in effective population size inferred by PSMC for three Iberian species.
Supplementary Fig. 26 Historical dynamics in effective population size inferred by PSMC for species of the Balkan species group.
Supplementary Fig. 27 Historical dynamics in effective population size inferred by PSMC for two lineages of *P. siculus*.

Supplementary Tables

Supplementary Table 1 Sample Information.
Supplementary Table 2 Methods for phylogenetic framework.
Supplementary Table 3 Information on mitochondrial genome assemblies.
Supplementary Table 4 Summary of all introgression events among *Podarcis* species.
Supplementary Table 5 Distribution of average dN/dS ratios for *Podarcis* lineages of genes.
Supplementary Table 6 Gene list in the genomic block (candidate region) on Chr. 15.
Supplementary Table 7 Permits for specimen collection.

Supplementary Information References

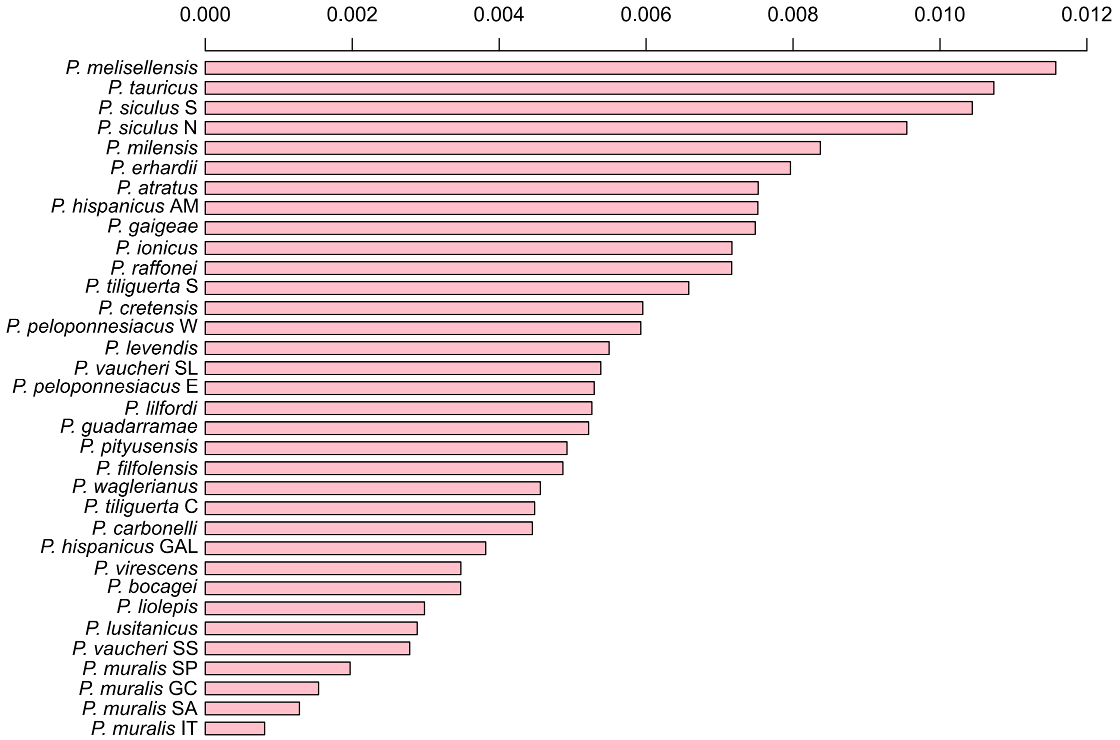

Supplementary Fig. 1 | Distribution of missing rate of SNVs for each *Podarcis* lineage. As expected, samples of the same species as the reference genome, namely *P. muralis*, have the lowest missing rate of SNVs. Overall, the average missing rate was less than 0.6%.

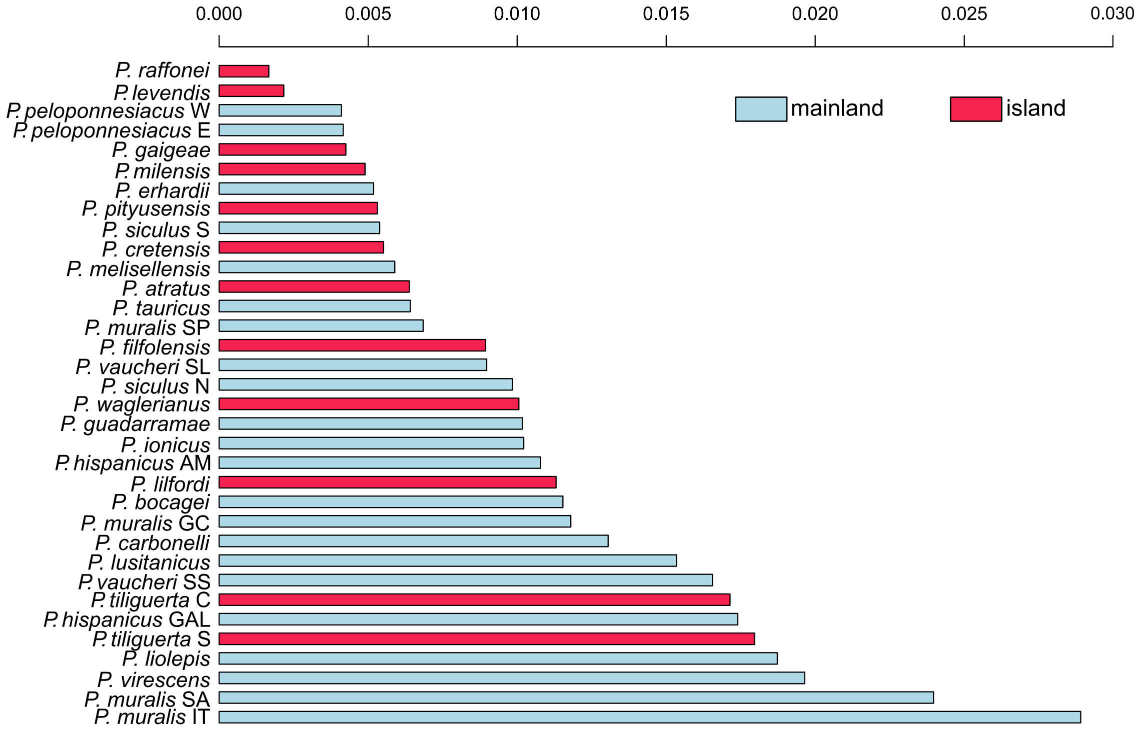

Supplementary Fig. 2 | Distribution of genetic diversity (π) for each *Podarcis* lineage. The blue bars indicate the mainland species, whereas the red bars represent the island species.

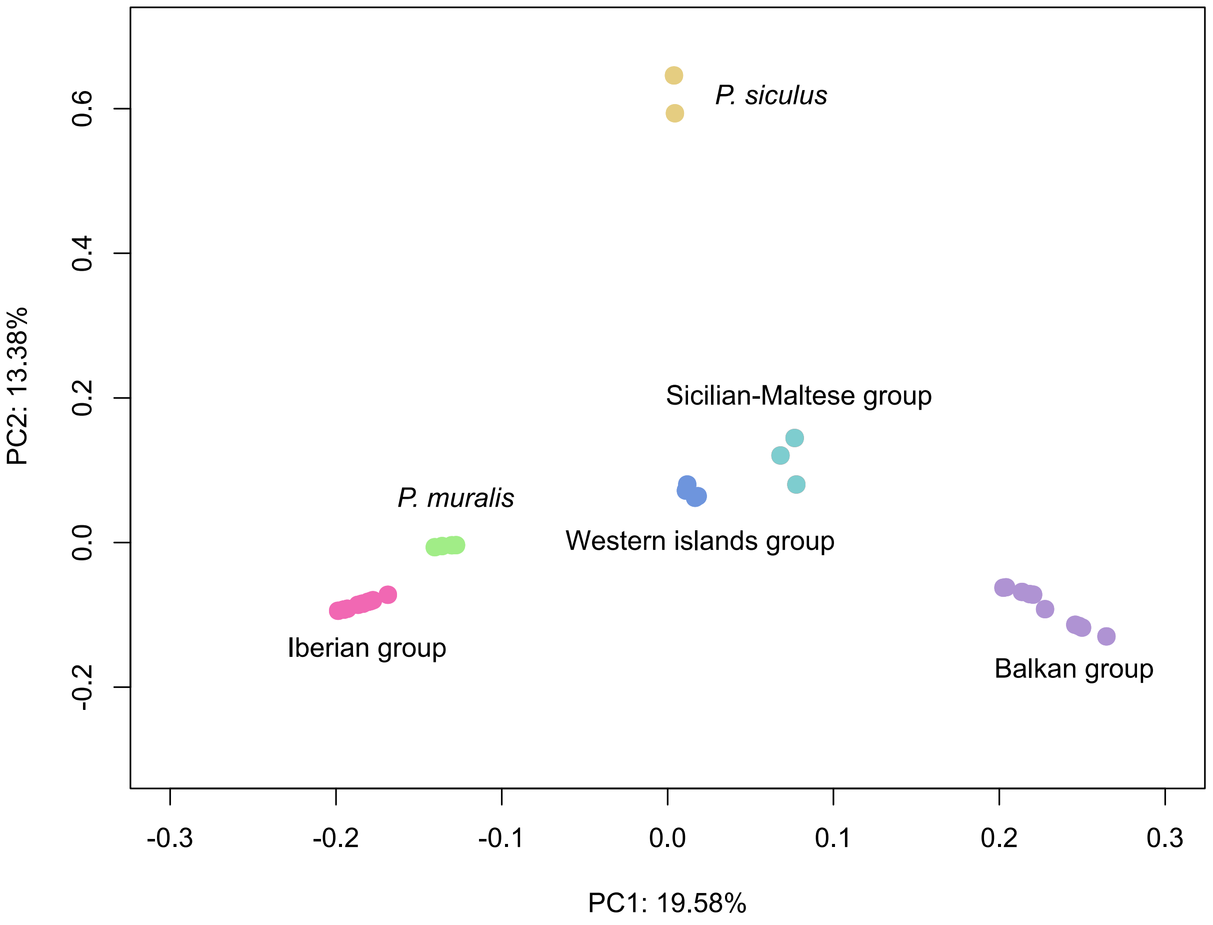

Supplementary Fig. 3 | PCA plot of genetic distance for all *Podarcis* lineages. Genetic distances were calculated based on WGS SNVs. All samples are clearly separated into groups according to their geographic distribution (except for *P. muralis* and *P. siculus* which form separate cluster.

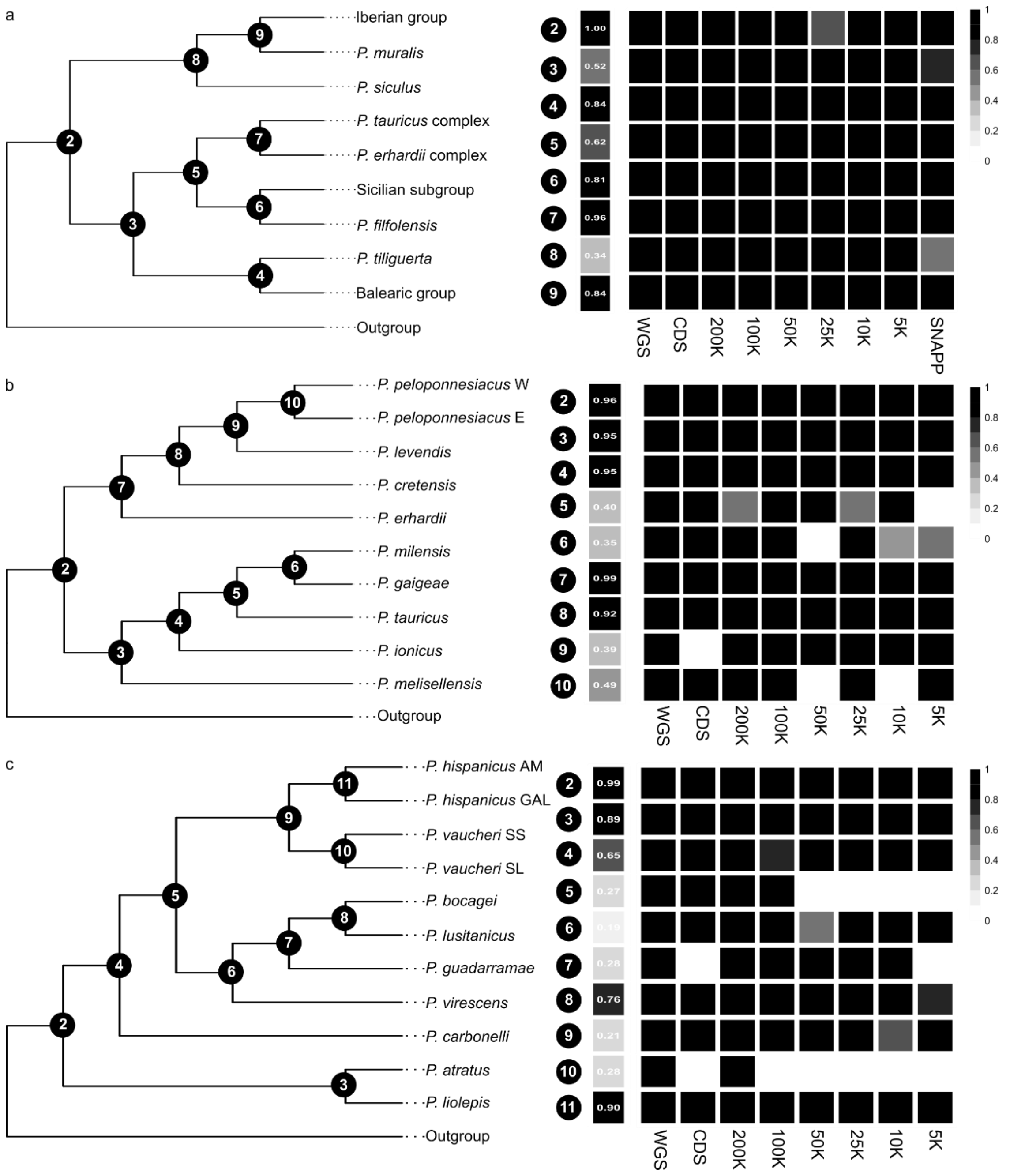

Supplementary Fig. 4 | Summary of node support across methodologies. Left panels show phylogenetic trees of **a,** major clades of *Podarcis*, **b,** the Balkan group and **c,** the Iberian group. Black circles with numbers represent the nodes on the phylogeny. Right panels show the support rate based on different datasets and methods for each node. The first column with numbers in the squares is the proportion of local trees derived from 200-kb windows that support the nodes. The grey-scale of squares in the following columns represent bootstrap values (lighter shade indicates a lower support). A blank square indicates that the node is not supported by the corresponding phylogeny.

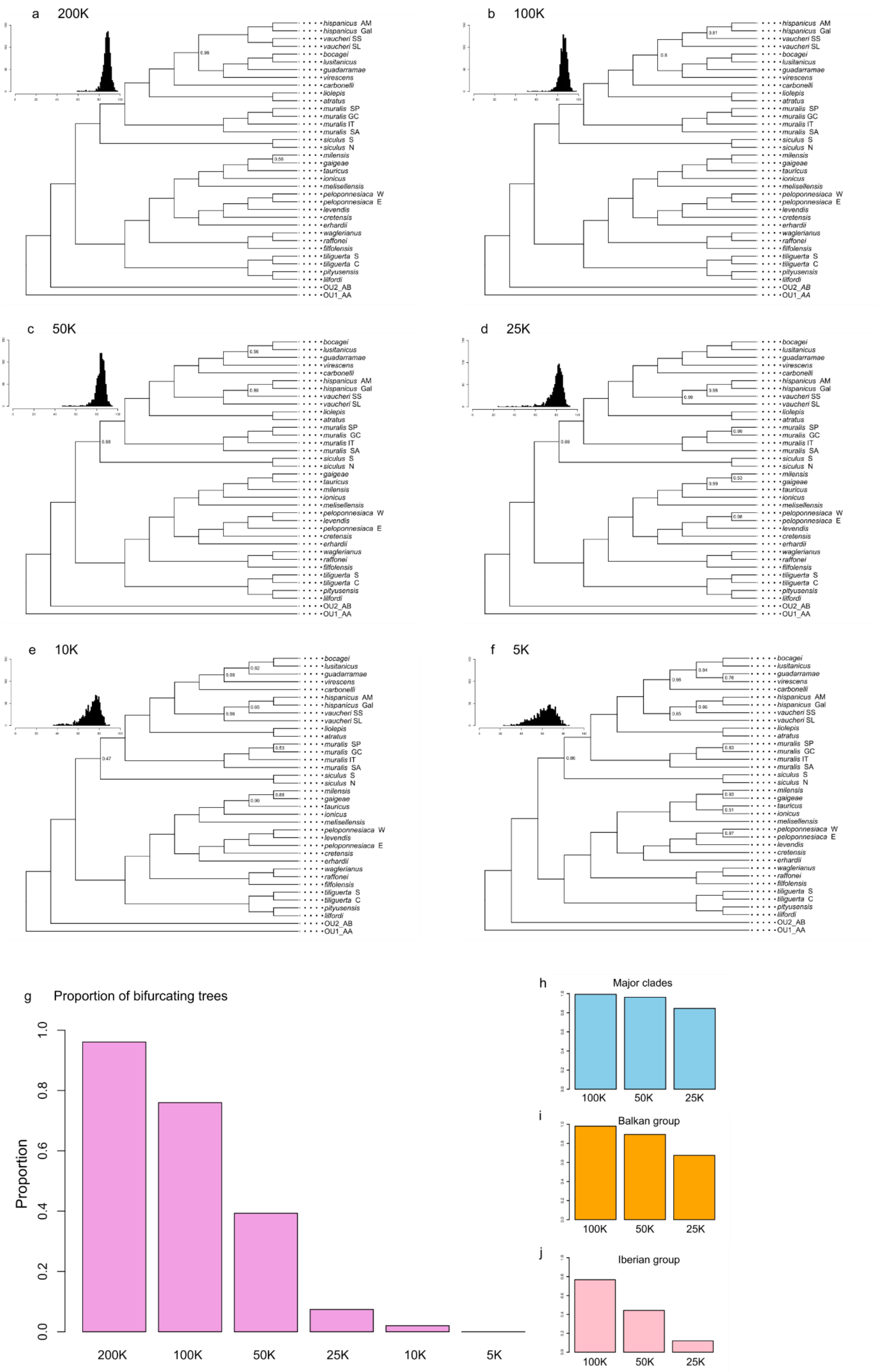
a 200K
hispanicus AM
hispanicus Gal
vaucheri SS
vaucheri SL
bocagei
lusitanicus
guadarramae
virescens
carbonelli
liolepis
atratus
muralis SP
muralis GC
muralis IT
muralis SA
siculus S
siculus N
milensis
gaigeae
tauricus
ionicus
melisellensis
peloponnesiaca W
peloponnesiaca E
levendis
cretensis
erhardii
waglerianus
raffonei
filfolensis
tiliguerta S
tiliguerta C
pityusensis
lilfordi
OU2_AB
OU1_AA
b 100K
c 50K
d 25K
e 10K
f 5K
g Proportion of bifurcating trees
Proportion
200K
100K
50K
25K
10K
5K
h Major clades
i Balkan group
j Iberian group

Supplementary Fig. 5 | Consistency of topologies among different window sizes. **a-f,** The *Podarcis* trees inferred by multi-species coalescent approach (ASTRAL) from fixed-window local trees with different lengths **a,** 200 kb, **b,** 100 kb, **c,** 50 kb, **d,** 25 kb, **e,** 10 kb, and **f,** 5 kb. Bayesian posterior probabilities are provided next to the nodes unless they were 1. Regardless of window size, all topologies are highly consistent. Each panel shows a frequency distribution of bootstrap values on the top left. **g-j,** The proportion of fully resolved local trees (i.e., bifurcating tree) inferred from the different window sizes. The proportion decreases rapidly from 100 kb to 25 kb. However, most local trees are bifurcating for branches leading to **h,** major clades and **i,** within the Balkan group. Most polytomies are located in the **j,** Iberian group.

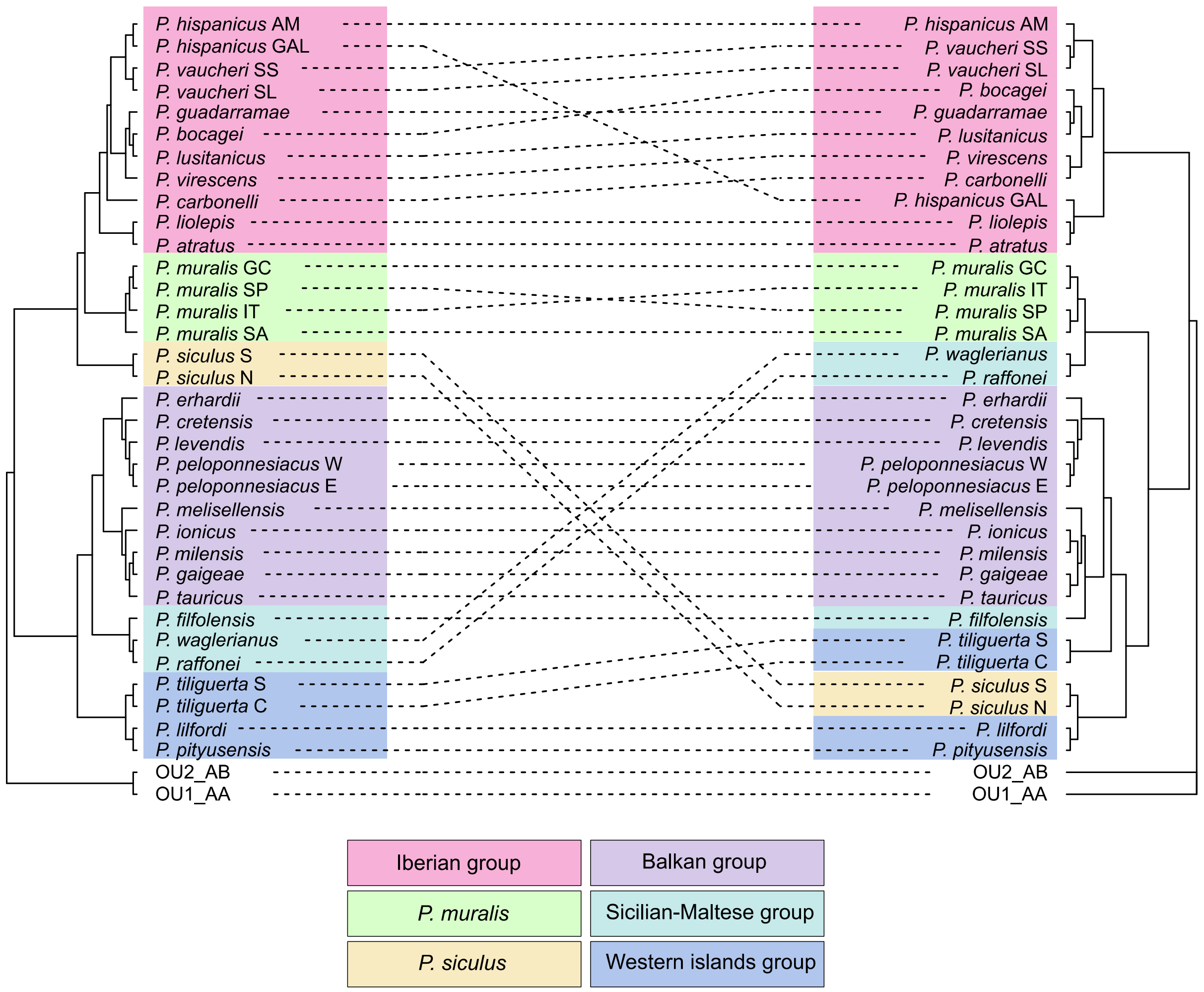

Supplementary Fig. 6 | Discrepancy between nuclear and mitochondrial trees. The discrepancies between phylogenies based on concatenated WGS SNVs (left) and on mitochondrial genome data (right).

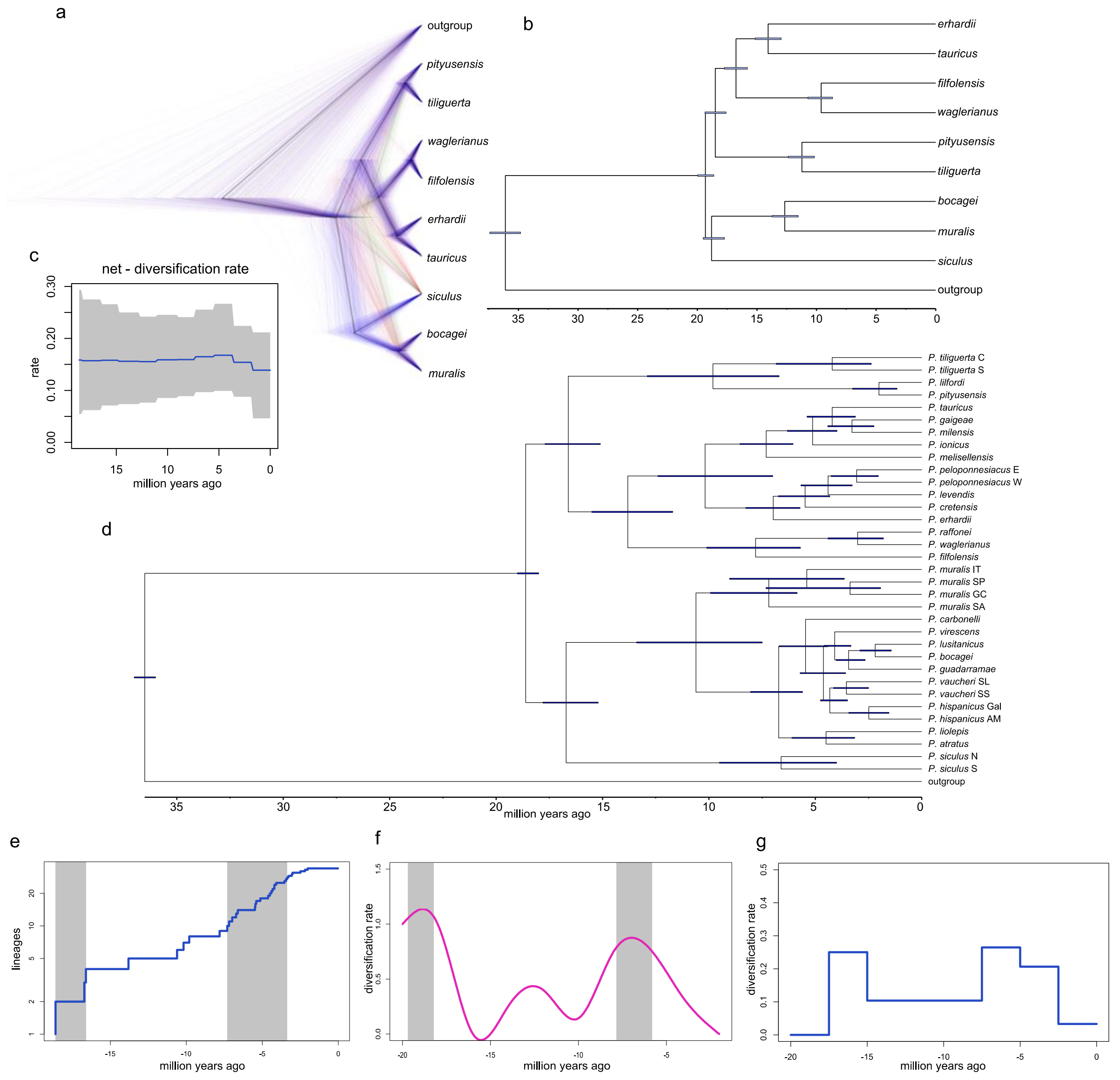

Supplementary Fig. 7 | Timing of diversifications. a, A 'DensiTree' illustration of the phylogenies inferred by SNAPP in the BEAST2 software including ten species. Each line in this plot represents a tree sample from every 1000th MCMC iteration of the run. The consensus tree is shown with a grey thick line. **b,** The consensus tree of SNAPP inference for the *Podarcis* tree. The blue bars represent the 95% credibility for the timing of a split. Due to computational limitations, only a subset of species were selected. The topology is identical to the major clades from both concatenating and multispecies coalescent methods. **c,** Estimation of diversification rate by RevBayes. The effect is weak but statistically supports two shifts of diversification rate. **d,** Time-calibrated tree inferred by MCMCtree. The blue bars represent the 95% credible intervals of estimated divergence times. **e,** Lineage Through Time (LTT) plot for all extant *Podarcis* species and lineages. The plot is based on the whole-genome time-calibrated tree. The dash lines indicate the 95% CI of the lineages. The LTT plot suggests that the number of lineages increases during ca 17-15 million years ago (MYA) and 6.5-4.0 MYA. **f,** Estimation of diversification rate by using a sliding window.

The window size is 2 million years with steps of 0.5 million years. The result is consistent with the LTT plot in panel **e**. The two grey-shaded, vertical bands in panels **e** and **f** represent the time interval in which the diversification rates were higher than average. **g,** Estimation of diversification rate by maximum likelihood method in treePar. The result also supports two shifts in diversification rate across the time-calibrated tree. The results shown in panels **c** to **g** are based on selected genomic regions whole local trees were concordant with the consensus phylogeny.

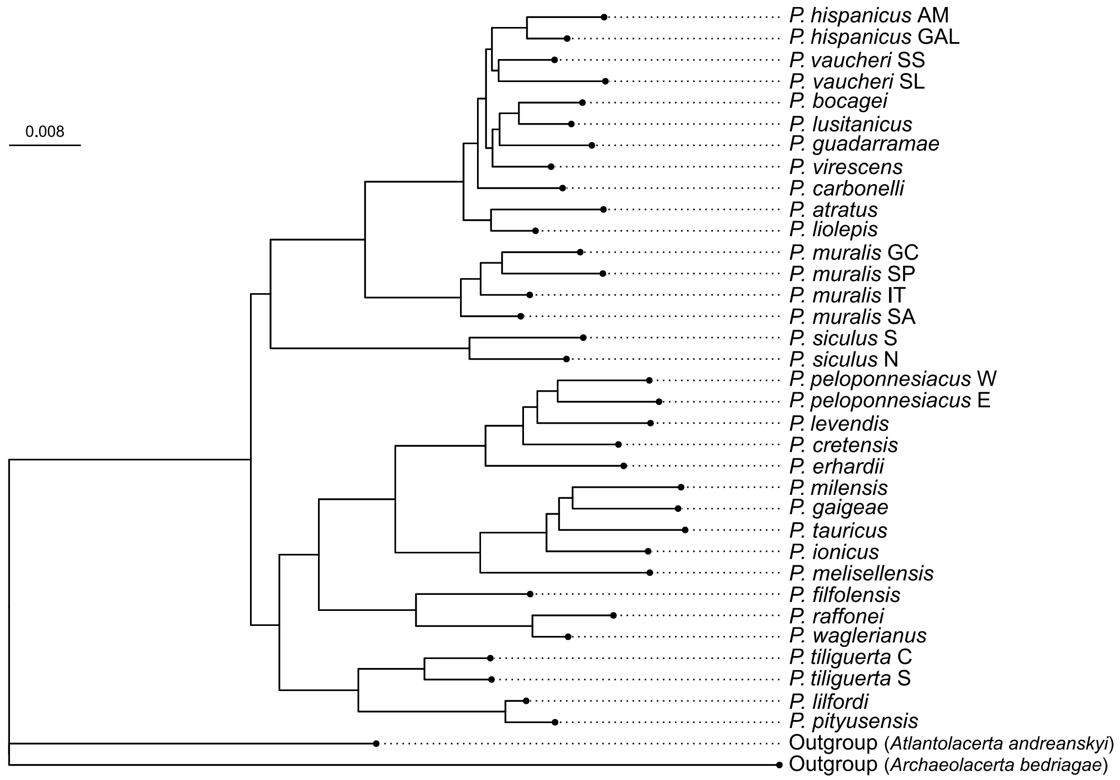

Supplementary Fig. 8 | Maximum-likelihood (ML) tree of *Podarcis* lizards based on the concatenating SNVs across the whole genome (WGS dataset). The bootstrap values for all branches are 100. The phylogeny clearly split all species into two clades: with *P. siculus*, *P. muralis*, and Iberian group belonging to clade 1; Mediterranean islands species (except for *P. atratus*) and Balkan group belonging to clade 2.

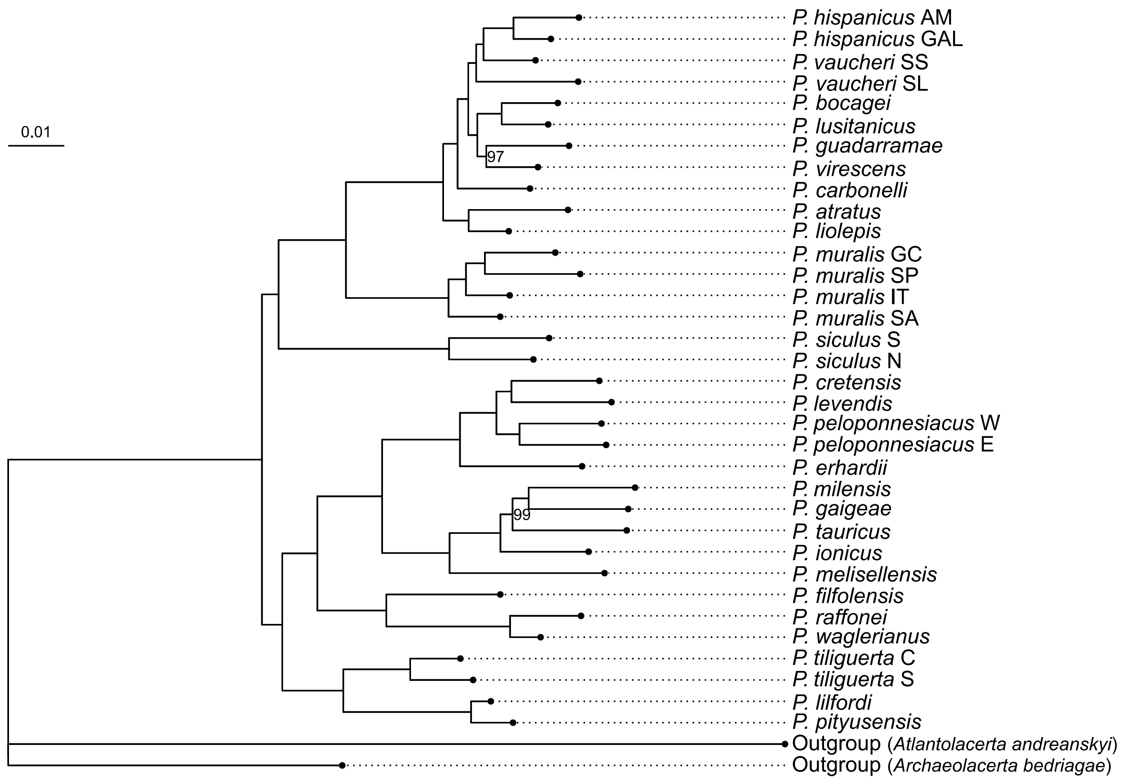

Supplementary Fig. 9 | Maximum-likelihood (ML) tree based on only SNVs from sequences of protein-coding genes (CDS dataset). The topology is similar to the whole-genome ML tree with minor discrepancies for *P. vaucheri* and *P. peloponnesiacus*. Bootstrap values are provided next to the nodes unless they were 100.

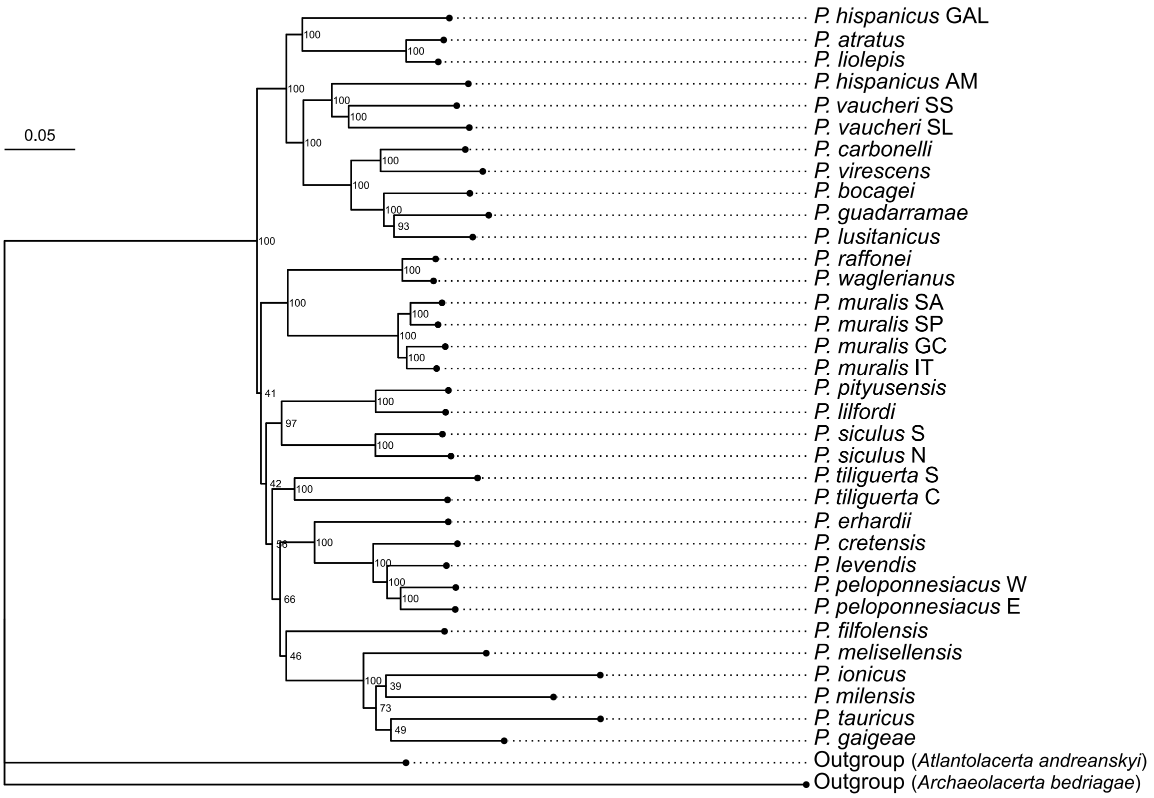

Supplementary Fig. 10 | Maximum-likelihood tree inferred from the mitochondrial genome (mtDNA dataset). The numbers next to the nodes represent bootstrap values. In this phylogeny, *P. waglerianus* and *P. raffonei* form a sister clade to *P. muralis*; and the Balearic species form a sister clade to *P. siculus* rather than *P. tiliguerta*. For discrepancies between this tree based on mitochondrial DNA and a tree based on nuclear DNA, see Supplementary Fig. 6.

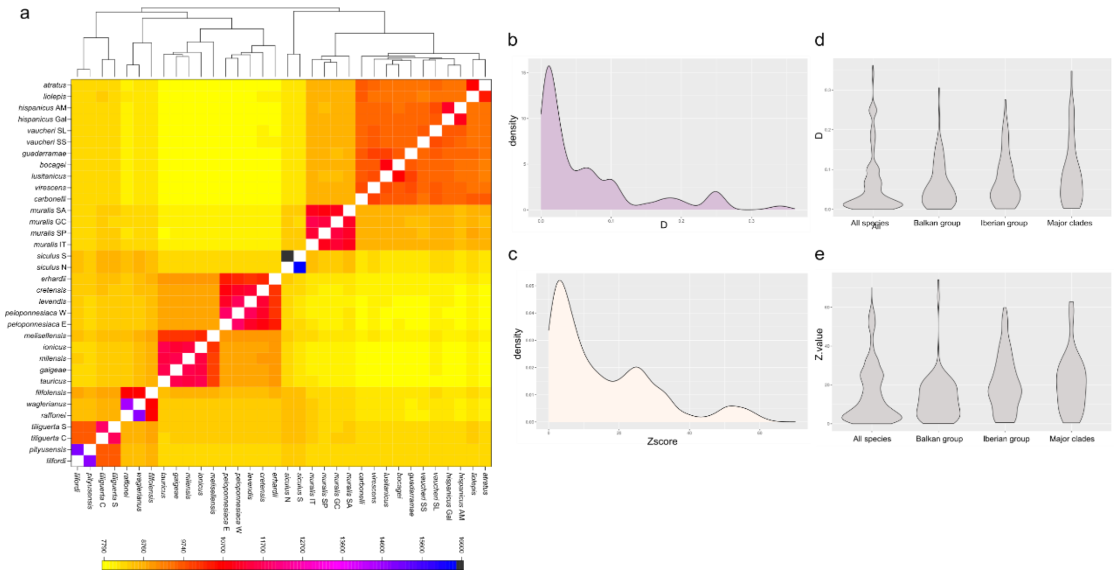

Supplementary Fig. 11 | Overview of admixture across *Podarcis*. a, Co-ancestry matrix of all *Podarcis* samples inferred by fineSTRUCTURE. The colour for each square represents the shared ancestry between two samples. **b, c,** Distribution of absolute values of D-statistic and Z-score for all triplet comparisons. The distribution indicates that the majority of triplets (77.02%) significantly deviate from neutrality (|Zscore| > 3.3). This suggests that the pervasive discordance between local trees and the consensus tree is not only caused by incomplete lineage sorting (ILS). **d, e,** Distribution of D-statistics for triplets from all samples, major clades, Iberian and Balkan groups.

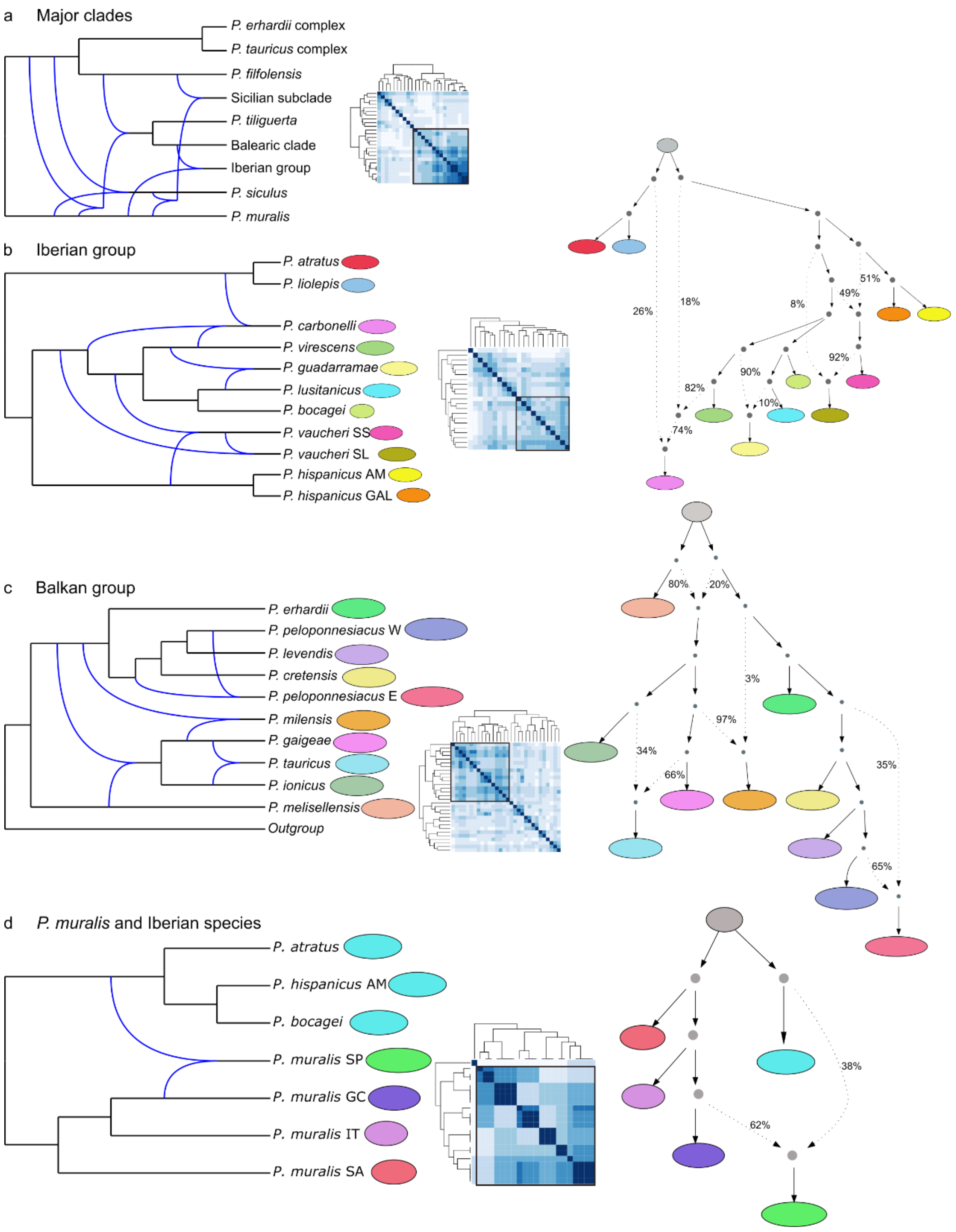

Supplementary Fig. 12 | Introgression events revealed by qpGraph and phyloNet. a-d, Left panel shows PhyloNet topologies for **a,** the major clades, **b,** the Iberian group, **c,** the Balkan group and **d,** *P. muralis* and the Iberian species. The black lines indicate tree-like evolution, while the blue lines indicate reticulation events. Right to the PhyloNet topologies are symmetrical matrices, and each row or column represents the most likely network in a single run of PhyloNet MCMC_gt. The consensus network shown to the left was inferred

based on the networks within the area of the matrix that is framed by a black box. To the right of each panel are qpGraphs based on the same taxa and dataset as shown in the PhyloNet topologies to the left. The black lines indicate tree-like evolution, while the dashed lines indicate reticulation events. Note that the qpGraph of panel a is omitted here as it is shown in Fig. 3b. Colour-coded ovals show correspondence of taxa between phyloNet and qpGraph. For model tests of all introgression events, see Supplementary Figs. 14-21.

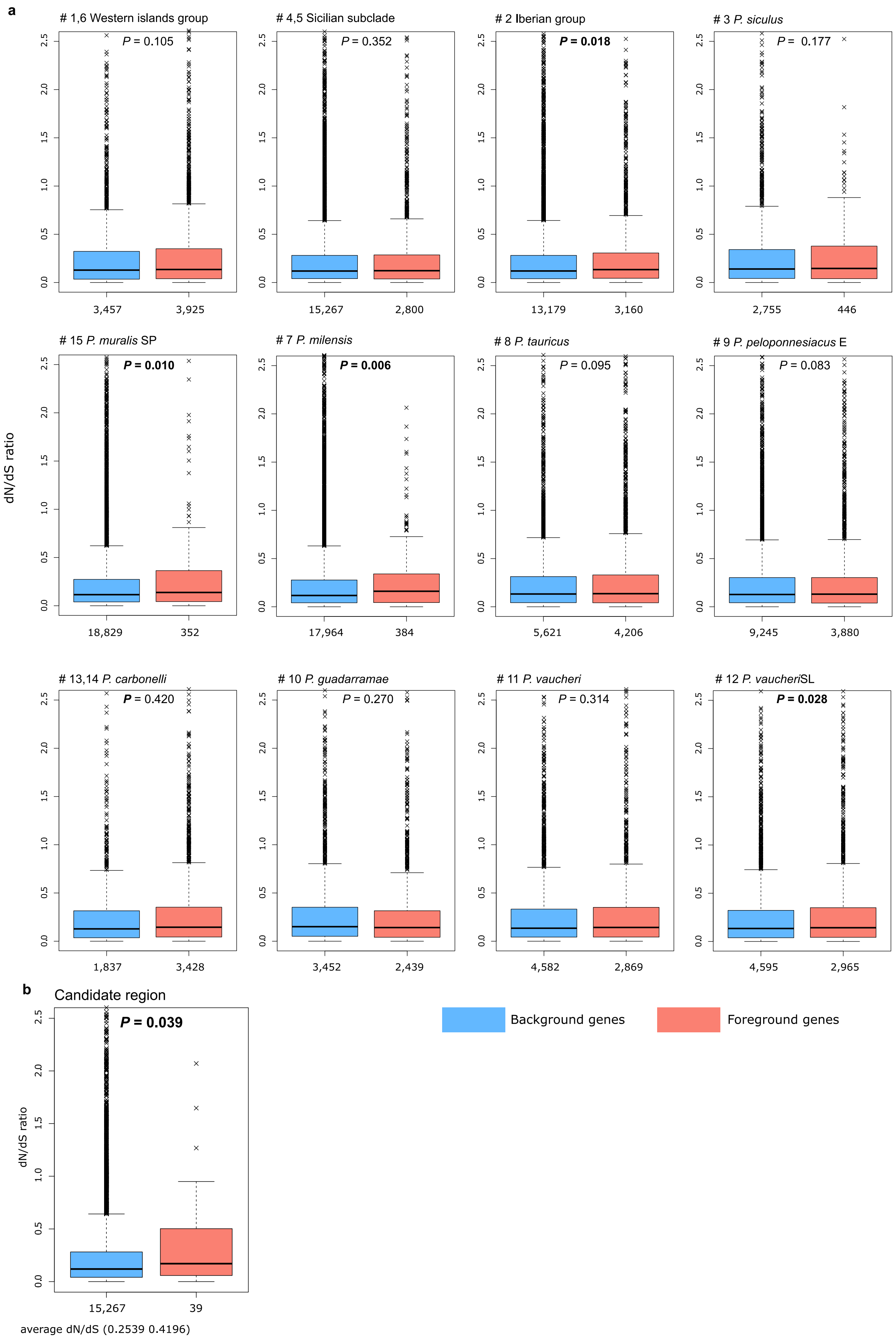
a
# 1,6 Western islands group
P = 0.105
3,457
3,925
# 4,5 Sicilian subclade
P = 0.352
15,267
2,800
# 2 Iberian group
P = 0.018
13,179
3,160
# 3 P. siculus
P = 0.177
2,755
446
dN/dS ratio
# 15 P. muralis SP
P = 0.010
18,829
352
# 7 P. milensis
P = 0.006
17,964
384
# 8 P. tauricus
P = 0.095
5,621
4,206
# 9 P. peloponnesiacus E
P = 0.083
9,245
3,880
# 13,14 P. carbonelli
P = 0.420
1,837
3,428
# 10 P. guadarramae
P = 0.270
3,452
2,439
# 11 P. vaucheri
P = 0.314
4,582
2,869
# 12 P. vaucheriSL
P = 0.028
4,595
2,965
b
Candidate region
P = 0.039
dN/dS ratio
15,267
39
average dN/dS (0.2539 0.4196)
Background genes
Foreground genes

Supplementary Fig. 13 | Pattern of protein-coding gene evolution measured by dN/dS ratio. a, Comparison of dN/dS ratios between protein-coding genes that are derived from introgression events and those without a history of introgression (background genes) for the 12 hybrid lineages. The number of genes per group are given below each plot. Significance levels for each comparison are given on top of each panel and were derived from 1,000 permutations. **b,** dN/dS ratio of the 39 genes located in the candidate region in the Sicilian subclade. For a summary of the estimated dN/dS ratios, see Supplementary Table 5. In both panels a and b, boxes represent interquartile ranges and whiskers indicate the range that lies within 1.5 times of the interquartile ranges. Crosses show data points outside this range. Statistical tests (permutation tests) were one-sided and no adjustments for multiple comparisons were applied.

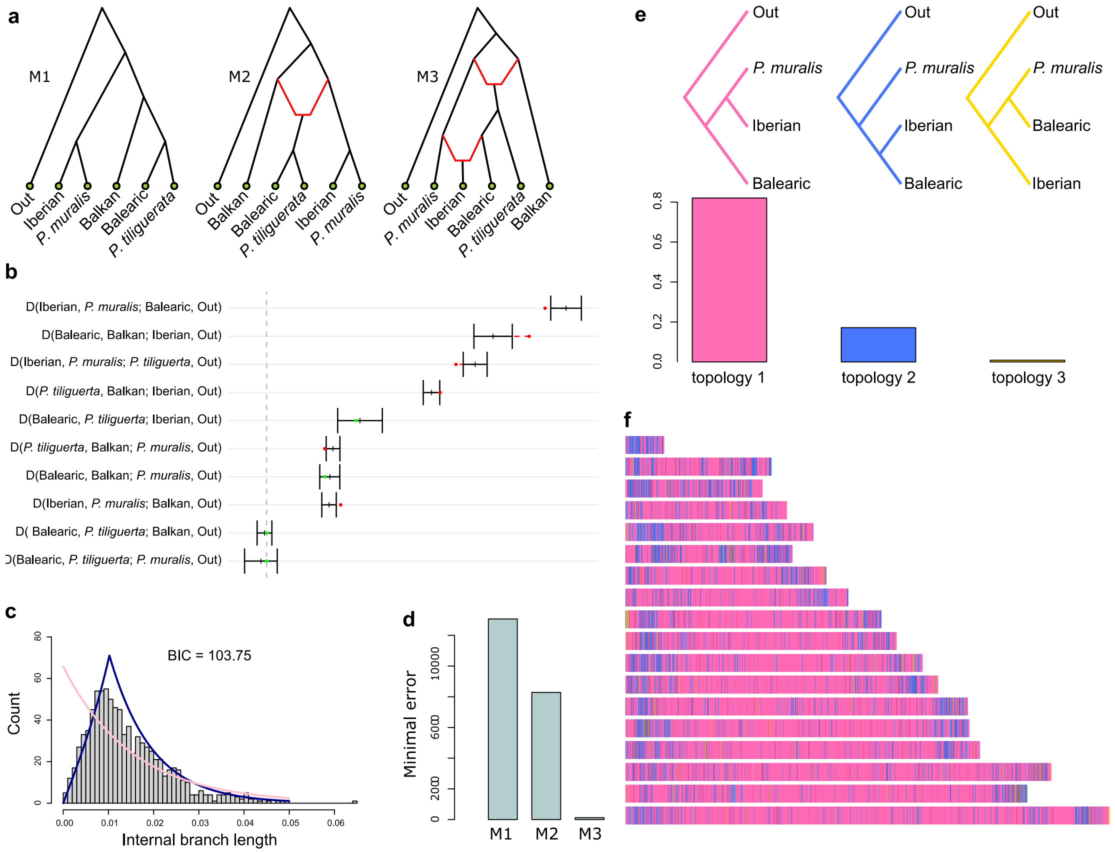

Supplementary Fig. 14 | Reticulate evolution between *P. muralis* - Iberian clade and Western Islands clade. a, Schematic diagram for introgression scenarios between the two clades: (M1) consensus tree with no introgression; (M2) an ancient introgression from the most recent common ancestor (MRCA) of *P. muralis* - Iberian clade into the MRCA of Western Islands; (M3) M2 plus a recent introgression from MRCA of Balearic species *(P. lilfordi* and *P. pityusensis*) into the MRCA of Iberian species. The red lines indicate the reticulations. **b,** D-statistics for the triplets from the species in panel a. Black error bars represent the standard deviation of the observed D-statistic. Solid, coloured dots represent the expected D-values from the best-fitting scenario from admixturegraph (measure of goodness-of-fit): green dots indicate that the expected values are within the observed range, whereas red dots and dash lines indicate the deviation from observed data. **c,** The distribution of internal branch lengths between Iberian species and Balearic species and the corresponding model of ILS (pink line) and introgression (blue line) from QuIBL analysis. The introgression model fits better with BIC = 103.75. **d,** The minimal errors for the scenarios, in which M3 has the minimum value. **e,** The distribution of tree topologies between Balearic species and *P. muralis* - Iberian clade. **f,**

The distribution of tree topologies across the genome, where the colouration is consistent with panel .

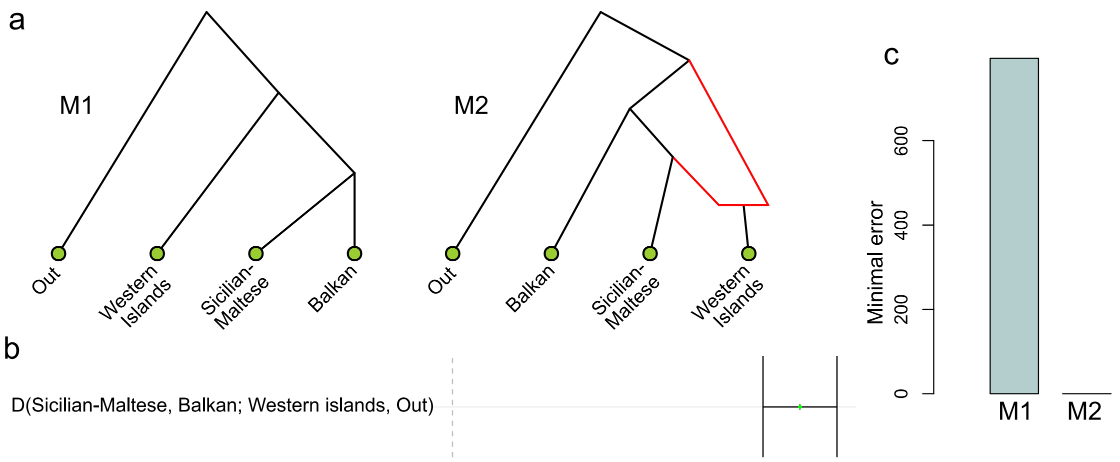

Supplementary Fig. 15 | Model test for introgression between Western Islands and Sicilian-Maltese groups. a, Schematic diagram for introgression scenarios between the two clades: (M1) species tree with no introgression; (M2) an ancient introgression from an ancient 'ghost' lineage into the MRCA of the Western Islands clade. **b,** D-statistics for the triplets from the species in panel a. Black error bars represent the standard deviation of the observed D-statistic. Solid, coloured dots represent the expected D-values from the best-fitting scenario from admixturegraph (measure of goodness-of-fit): green dots indicate that the expected values are within the observed range, whereas red dots and dash lines indicate the deviation from observed data. **c,** The minimal errors for the scenarios, in which M2 has the minimum value.

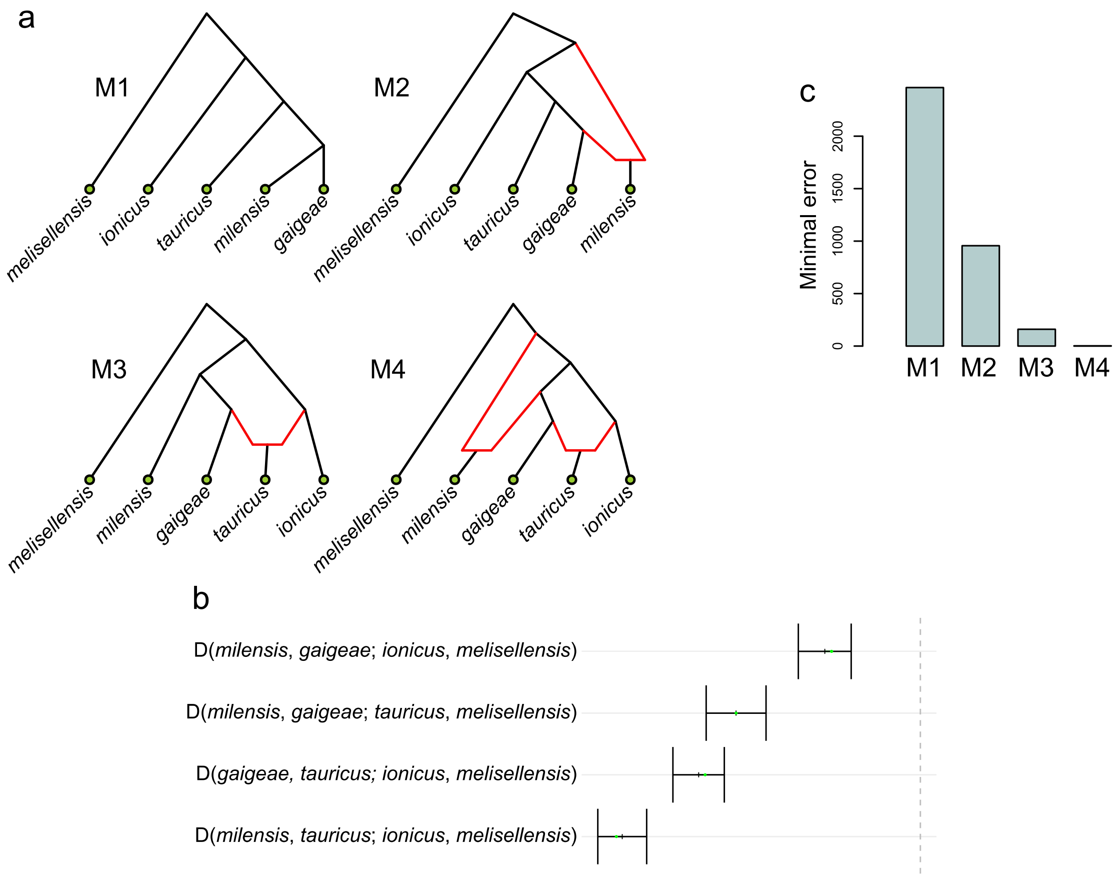

Supplementary Fig. 16 | Model test for introgression in *P. tauricus* species complex. a, Schematic diagram for introgression scenarios for *P. tauricus species* complex: (M1) species tree with no introgression; (M2) Introgression from an ancient 'ghost' clade into *P. milensis*. (M3) Hybrid speciation of *P. tauricus* from *P. gaigeae* and *P. ionicus*. (M4) Both reticulations. **b,** D-statistics for the triplets from the species in panel a. Black error bars represent the standard deviation of the observed D-statistic. Solid, coloured dots represent the expected D-values from the best-fitting scenario from admixturegraph (measure of goodness-of-fit): green dots indicate that the expected values are within the observed range, whereas red dots and dash lines indicate the deviation from observed data. **c,** The minimal errors for the scenarios, in which M4 has the minimum value.

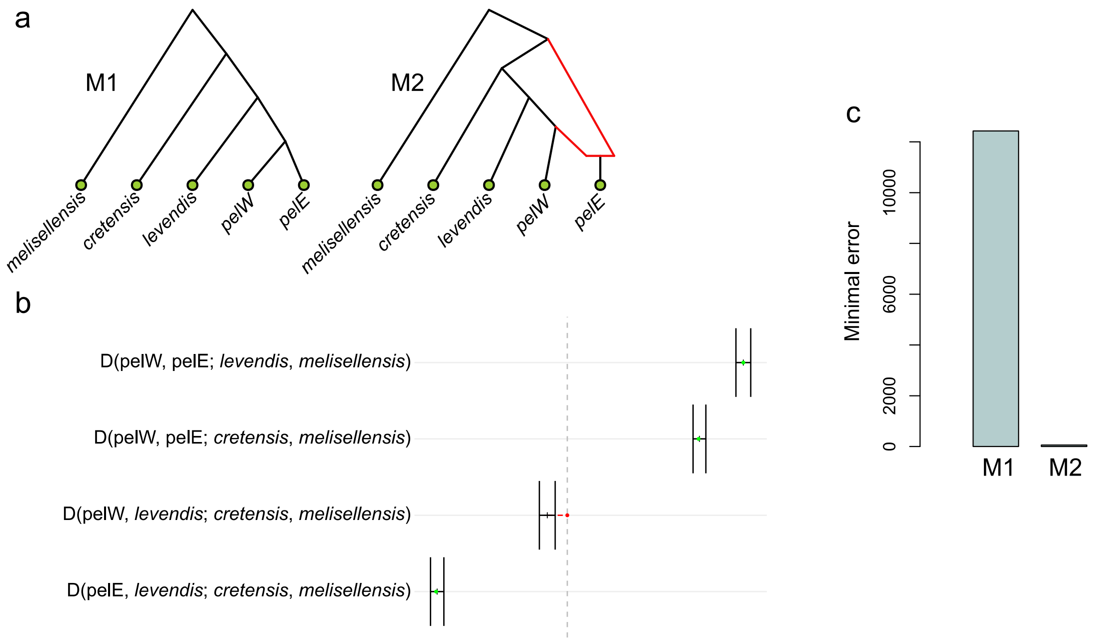

Supplementary Fig. 17 | Model test for introgression of the eastern lineage of *P. peloponnesiacus*. a, Schematic diagram for introgression scenarios between the two clades: (M1) species tree with no introgression; (M2) introgression from an ancient 'ghost' lineage into the eastern lineage of *P. peloponnesiacus*. **b,** D-statistics for the triplets from the species in panel a. Black error bars represent the standard deviation of the observed D-statistic. Solid, coloured dots represent the expected D-values from the best-fitting scenario from admixturegraph (measure of goodness-of-fit): green dots indicate that the expected values are within the observed range, whereas red dots and dash lines indicate the deviation from observed data. **c,** The minimal errors for the scenarios, in which M2 has the minimum value. Abbreviations: pelE, *P. peloponnesiacus* E; pelW, *P. peloponnesiacus* W.

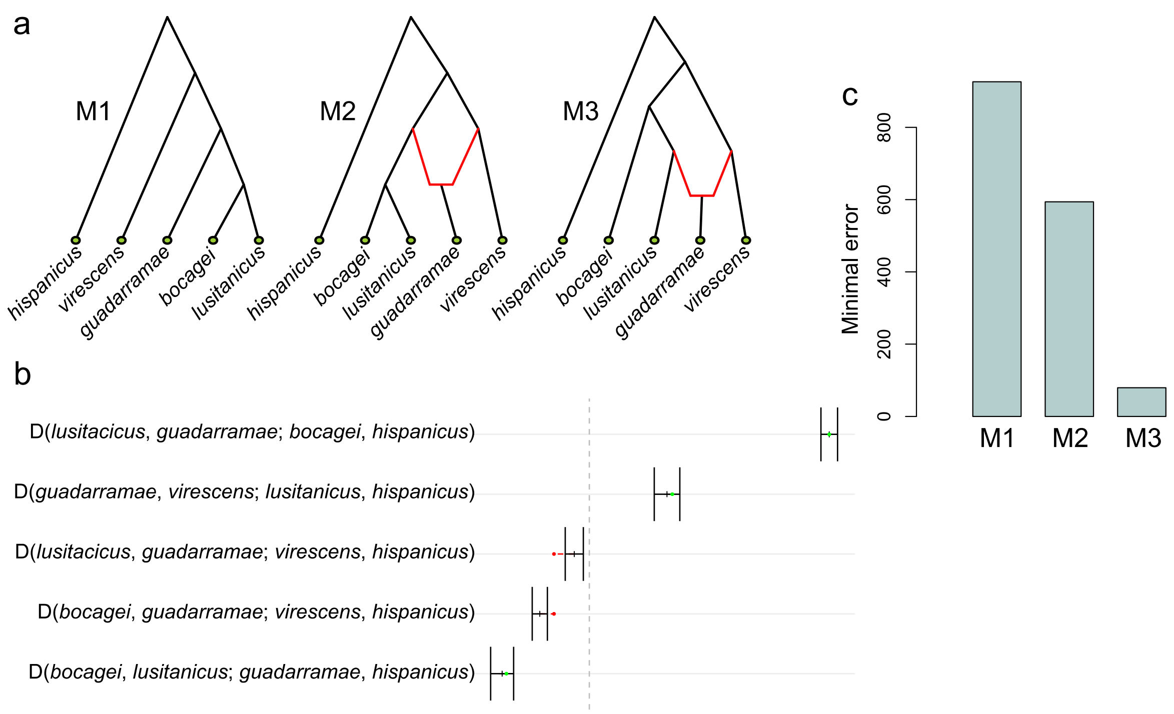

Supplementary Fig. 18 | Model test for hybrid origin of *P. guadarramae*. a, Schematic diagram for introgression scenarios between the two clades: (M1) species tree with no introgression; (M2) hybrid origin of *P. guadarramae* from *P. virescens* and the MRCA of *P. bocagei* and *P. lusitanicus*; (M3) hybrid origin of *P. guadarramae* from *P. virescens* and *P. lusitanicus*. **b,** D-statistics for the triplets from the species in panel a. Black error bars represent the standard deviation of the observed D-statistic. Solid, coloured dots represent the expected D-values from the best-fitting scenario from admixturegraph (measure of goodness-of-fit): green dots indicate that the expected values are within the observed range, whereas red dots and dash lines indicate the deviation from observed data. **c,** The minimal errors for the scenarios, in which M3 has the minimum value.

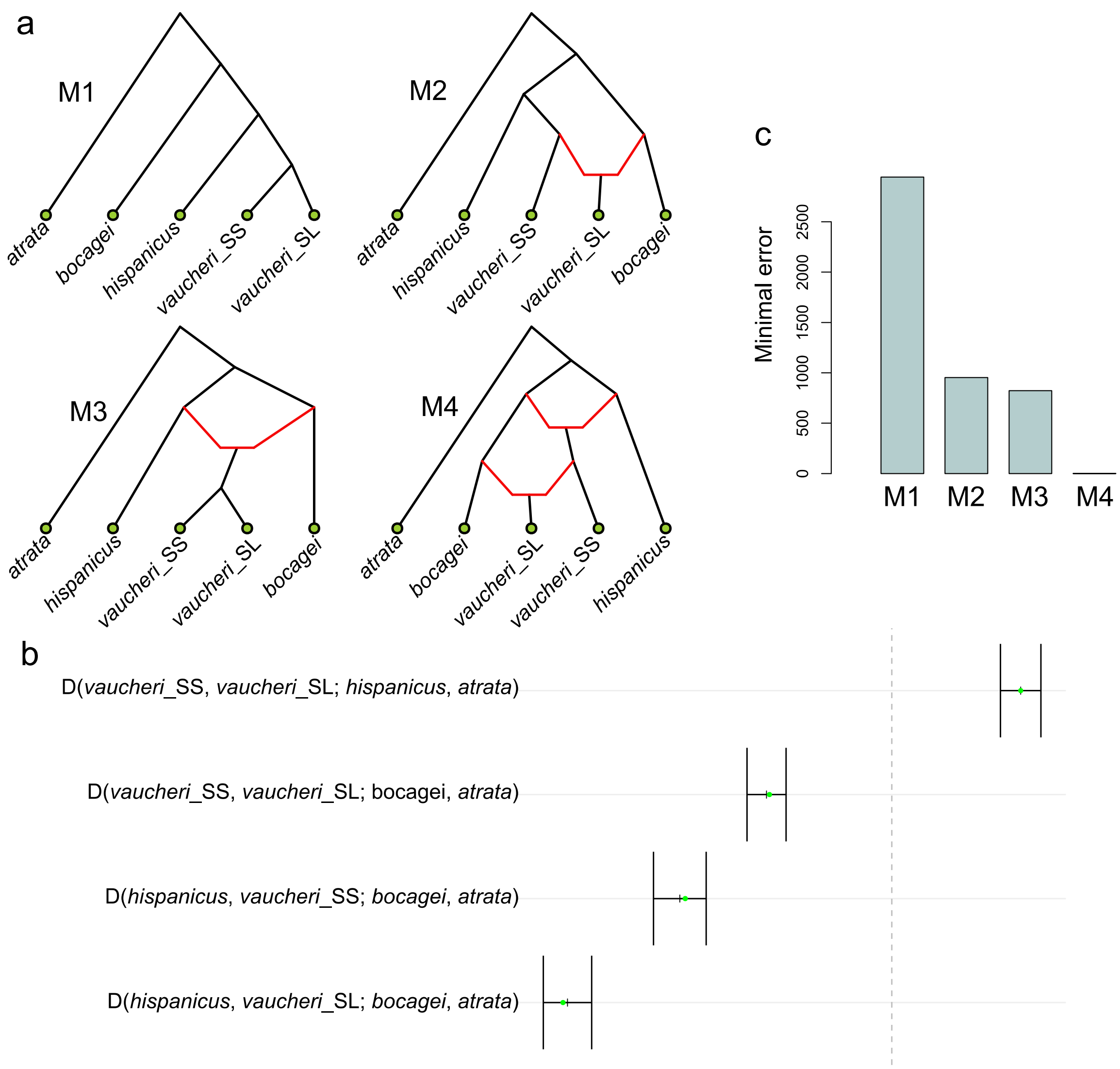

Supplementary Fig. 19 | Model test for reticulate evolution of *P. vaucheri*. a, Schematic diagram for introgression scenarios between the two clades: (M1) species tree with no introgression; (M2) an ancient introgression from the MRCA of *P. bocagei*, *P. guadarramae*, *P. lusitanicus*, and *P. virescens*, into the MRCA of *P. vaucheri*; (M3) a recent introgression from the same provider but into the "sensu latu" lineage of *P. vaucheri*; (M4) both reticulations. **b,** D-statistics for the triplets from the species in panel a. Black error bars represent the standard deviation of the observed D-statistic. Solid, coloured dots represent the expected D-values from the best-fitting scenario from admixturegraph (measure of goodness-of-fit): green dots indicate that the expected values are within the observed range, whereas red dots and dash lines indicate the deviation from observed data. **c,** The minimal errors for the scenarios, in which M4 has the minimum value.

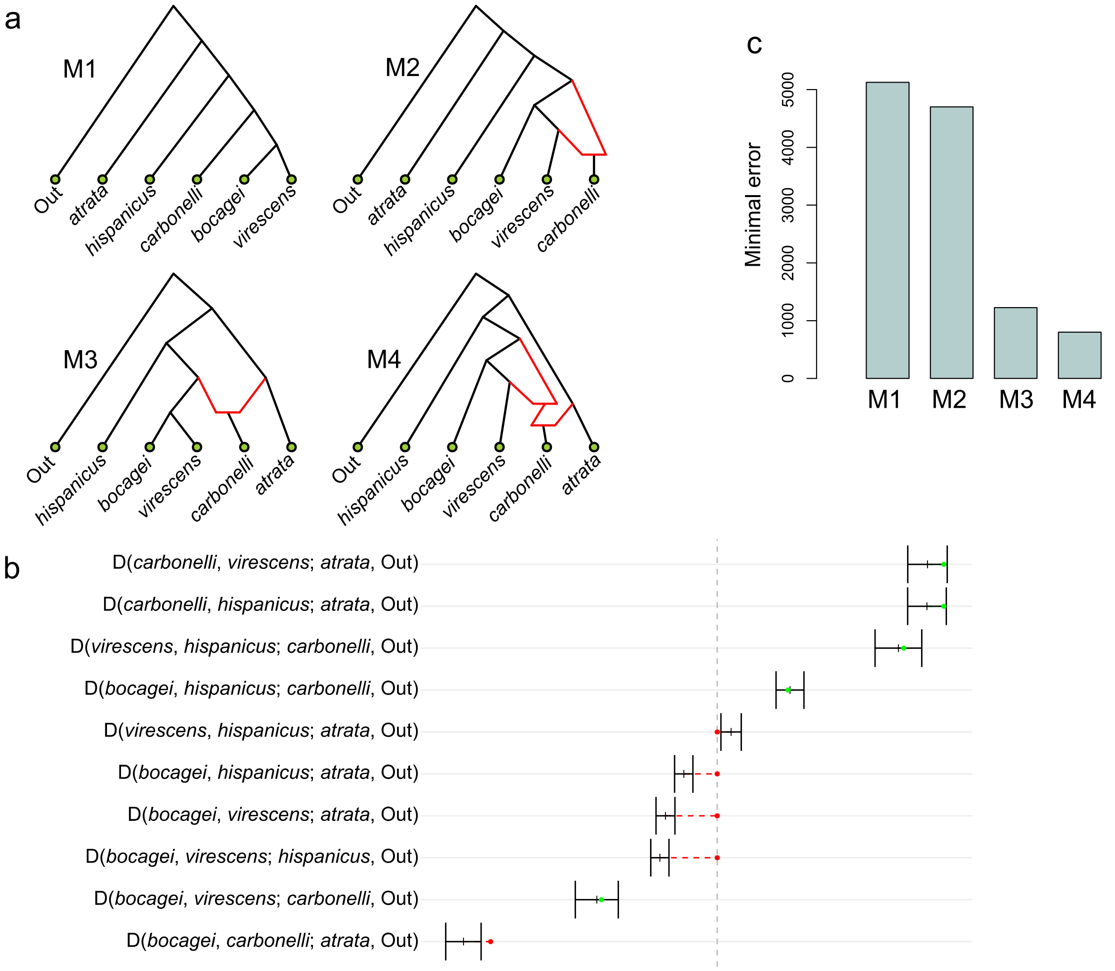

Supplementary Fig. 20 | Model test for reticulate evolution of *P. carbonelli.* **a,** Schematic diagram for introgression scenarios between the two clades: (M1) species tree with no introgression; (M2) introgression from *P. virescens* into *P. carbonelli*; (M3) introgression from the basal clade leading to *P. atratus* and *P. liolepis* into *P. carbonelli*; (M4) both reticulations. **b,** D-statistics for the triplets from the species in panel a. Black error bars represent the standard deviation of the observed D-statistic. Solid, coloured dots represent the expected D-values from the best-fitting scenario from admixturegraph (measure of goodness-of-fit): green dots indicate that the expected values are within the observed range, whereas red dots and dash lines indicate the deviation from observed data. **c,** The minimal errors for the scenarios, in which M4 has the minimum value; however, the high value also indicates cryptic admixture among the species.

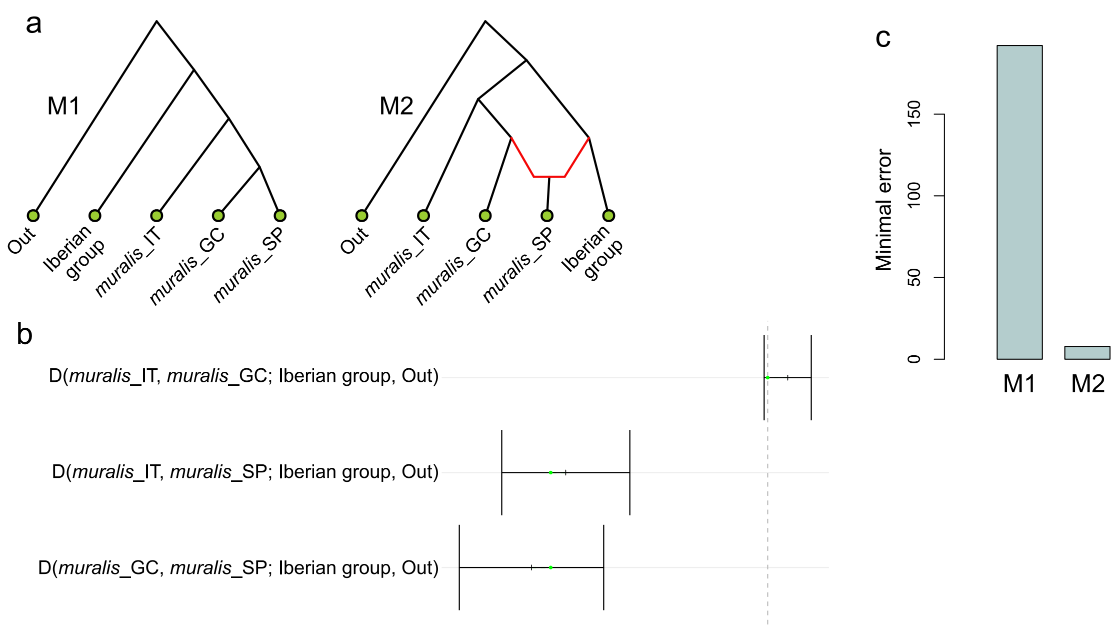

Supplementary Fig. 21 | Introgression for Spanish lineage of *P. muralis*. a, Schematic diagram for introgression scenarios between the two clades: (M1) species tree with no introgression; (M2) introgression from the MRCA of Iberian species group into the Spanish lineage of *P. muralis*. **b,** D-statistics for the triplets from the species in panel a. Black error bars represent the standard deviation of the observed D-statistic. Solid, coloured dots represent the expected D-values from the best-fitting scenario from admixturegraph (measure of goodness-of-fit): green dots indicate that the expected values are within the observed range, whereas red dots and dash lines indicate the deviation from observed data. **c,** The minimal errors for the scenarios, in which M2 has the minimum value.

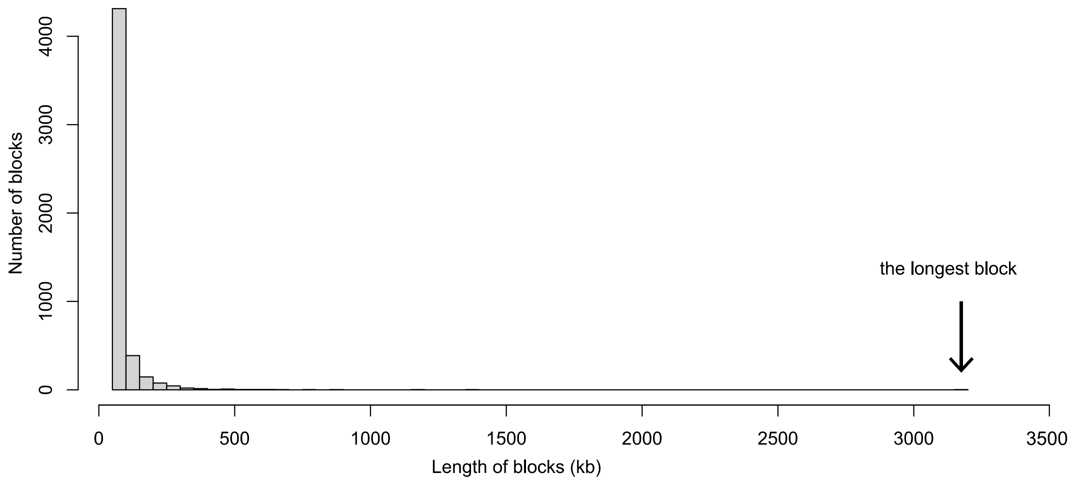

Supplementary Fig. 22 | Length distribution of genomic blocks for which *P. muralis* and the Sicilian subclade share the same ancestry. The arrow indicates the length of the longest block located on Chromosome 15.

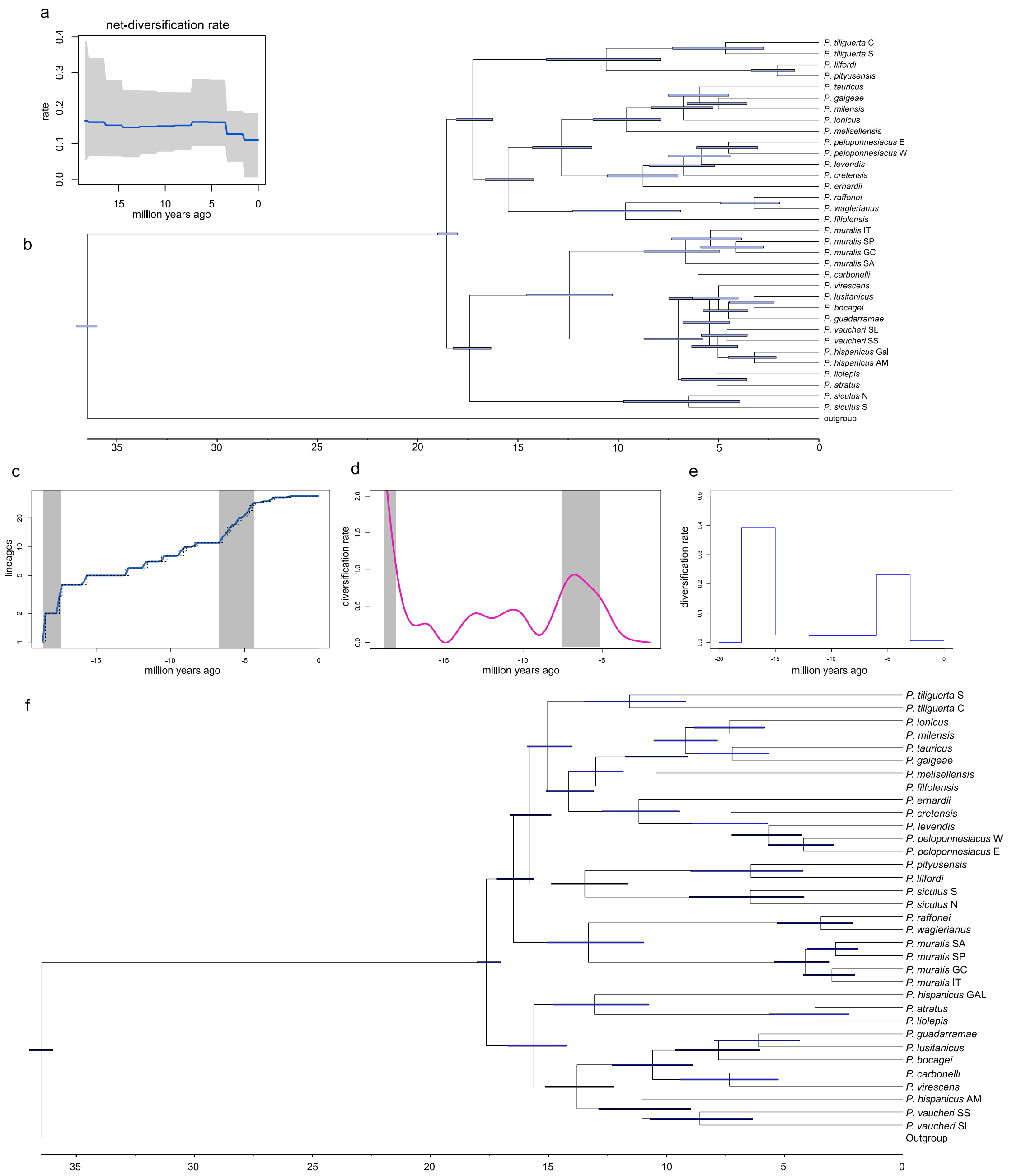

Supplementary Fig. 23 | Timing of diversifications based on the WGS dataset and on mitochondrial DNA. a, Estimation of diversification rate by RevBayes. The effect is weak but statistically supports two shifts of diversification rate. **b,** Time-calibrated tree inferred by MCMCtree. The blue bars represent the 95% credible intervals of estimated divergence times. **c,** Lineage Through Time (LTT) plot for all extant *Podarcis* species and lineages. The plot is based on the whole-genome time-calibrated tree. The dash lines indicate the 95% CI of the lineages. The LTT plot suggests that the number of lineages increases during ca 17-15 million years ago (MYA) and 6.5-4.5 MYA.

d, Estimation of diversification rate by using a sliding window. The window size is 2 million years with steps of 0.5 million years. The result is consistent with the LTT plot in panel **c**. The two grey-shaded, vertical bands in panels **c** and **d** represent the time interval in which the diversification rates were higher than average. **e,** Estimation of diversification rate by maximum likelihood method in treePar. The result also supports two shifts in diversification rate across the time-calibrated tree. **f,** Estimation of divergence times based on mitochondrial DNA. The results shown in panels **a** to **e** are based on the entire WGS dataset.

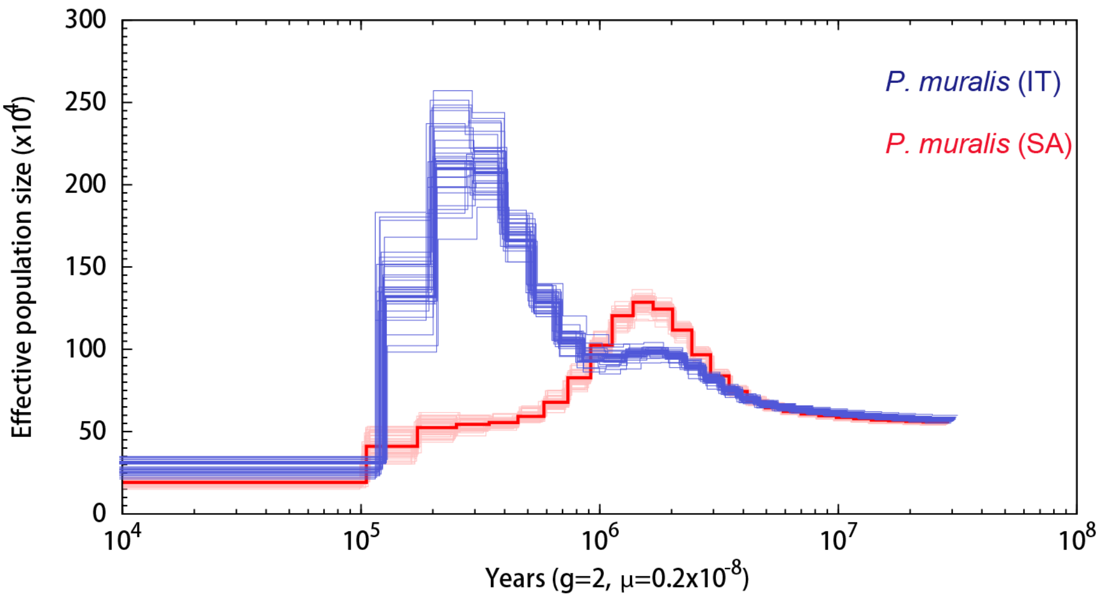

Supplementary Fig. 24 | Historical dynamics in effective population size inferred by PSMC for two lineages of *P. muralis*.

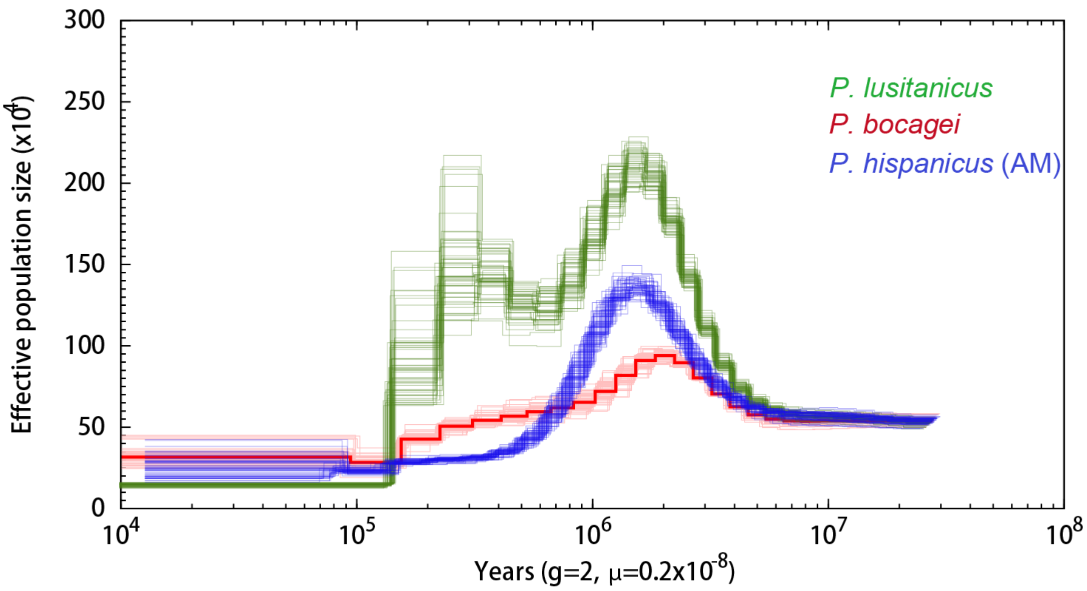

Supplementary Fig. 25 | Historical dynamics in effective population size inferred by PSMC for three Iberian species.

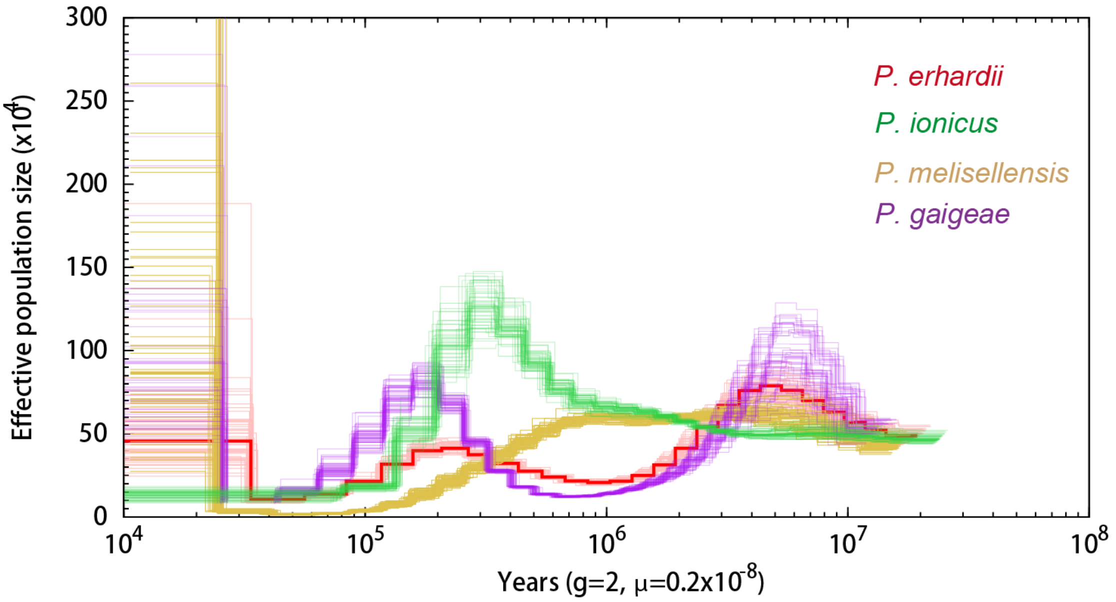

Supplementary Fig. 26 | Historical dynamics in effective population size inferred by PSMC for species of the Balkan species group.

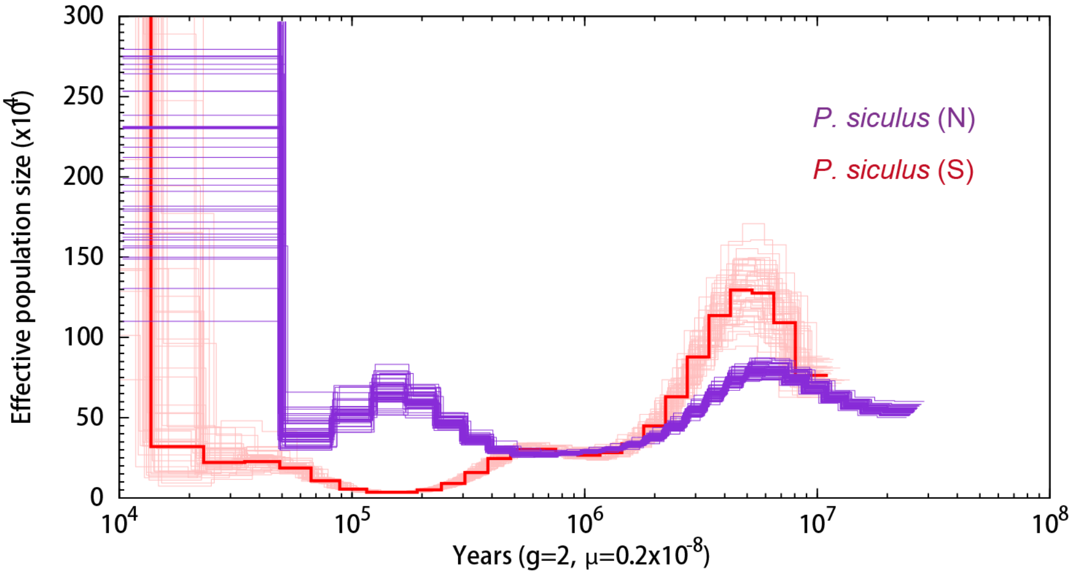

Supplementary Fig. 27 | Historical dynamics in effective population size inferred by PSMC for two lineages of *P. siculus*.

Supplementary Table 1 | Sample Information.

Sample_ID	Species	Latitude	Longitude	Note
Ab387.8	*Archaeolacerta bedriagae*	42.29	8.88	Outgroup
DB27608	*Atlantolacerta andreanskyi*	31.20	-7.87	Outgroup
DB5187	*Podarcis atratus*	39.875	0.67	potentially synonymized with *P. liolepis* but pending evaluation
PB01	*Podarcis bocagei*	41.30	-8.14	
DB19622	*Podarcis carbonelli*	40.77	-8.71	
DB28394	*Podarcis cretensis*	35.35	23.86	
DB28361	*Podarcis erhardii*	38.16	23.73	
2100-110	*Podarcis filfolensis*	35.96	14.42	
DB28481	*Podarcis gaigeae*	38.98	24.51	
DB22633	*Podarcis guadarramae*	40.67	-6.36	
DB7063	*Podarcis hispanicus*	38.76	-0.98	PHAM lineage (AM)[1]
DB7019	*Podarcis hispanicus*	37.60	-1.01	PHGal lineage (Gal); species status under debate[1,2]
DB28401	*Podarcis ionicus*	37.93	22.29	
DB28386	*Podarcis levendis*	36.02	23.25	
DB10319	*Podarcis lilfordi*	39.15	2.93	
DB24380	*Podarcis liolepis*	41.50	0.07	
DB22224	*Podarcis lusitanicus*	41.84	-8.87	species status under debate
DB20801	*Podarcis melisellensis*	45.51	13.90	
DB28514	*Podarcis milensis*	36.69	24.44	

DB13473	*Podarcis muralis*	40.37	-0.67	Iberian lineage (SP)[3]
M151	*Podarcis muralis*	39.18	21.73	Balkan lineage (GC)[3]
AS18	*Podarcis muralis*	42.41	13.51	Italian lineage (IT)[3]
NL24	*Podarcis muralis*	44.21	8.41	Southern Alps lineage (SA)[3]
DB28353	*Podarcis peloponnesiacus*	22.72	37.64	Eastern lineage (E)[4]
DB28371	*Podarcis peloponnesiacus*	37.93	22.29	Western lineage (W)[4]
DB13118	*Podarcis pityusensis*	38.95	1.45	
DB26218	*Podarcis raffonei*	38.82	15.25	
CAP3	*Podarcis siculus*	42.27	11.25	North lineage (N)[5]
TRC3	*Podarcis siculus*	38.03	12.32	South lineage (S)[5]
DB16305	*Podarcis tauricus*	41.96	27.39	
395A	*Podarcis tiliguerta*	42.43	9.33	Corsica lineage (C)[6]
PF2	*Podarcis tiliguerta*	41.25	9.23	Sardinia lineage (S)[6]
DB6975	*Podarcis vaucheri*	37.79	-3.78	*sensu stricto* (SS)[1]
DB25305	*Podarcis vaucheri*	32.04	-5.47	*sensu lato* (SL)[7]
DB19832	*Podarcis virescens*	38.57	-7.91	
DB26449	*Podarcis waglerianus*	37.93	12.31	

Supplementary Table 2 | Methods for phylogenetic framework.

Strategy	Dataset	Sites	Taxa	Method
Concatenation	Whole genome sequences (WGS)	28.41 M	36	ML
	Protein-coding sequences (CDS)	1.4 M	36	ML
	Mitochondrial genome (mtDNA)	13,854	36	ML
Multi-species coalescent (MSC)	200 Kb window	28.41 M	36	ML + ASTRAL
	100 Kb window	28.41 M	36	ML + ASTRAL
	50 Kb window	28.41 M	36	ML + ASTRAL
	25 Kb window	28.41 M	36	ML + ASTRAL
	10 Kb window	28.41 M	36	ML + ASTRAL
	5 Kb window	28.41 M	36	ML + ASTRAL
	Variants	3,000	10	SNAPP*
Divergence time estimation	Genomic regions concordant with consensus phylogeny	1.24 M	36	MCMCtree
	Whole genome sequencing (WGS)	28.41 M	36	MCMCtree

* SNAPP was only used to infer the relationships among major groups due to computational constraint.

Supplementary Table 3 | Information on mitochondrial genome assemblies.

ID	Sample tags	Contigs	Assembly*
A395	*tiliguerta*_C	2	all genes
AB387	OU2_AB	1	complete genome
AS18	*muralis*_IT	2	all genes
CAP3	*siculus*_N	2	all genes
DB10319	*lilfordi*	1	complete genome
DB13118	*pityusensis*	2	all genes
DB13473	*muralis*_SP	2	all genes
DB16305	*tauricus*	2	all genes
DB19622	*carbonelli*	2	all genes
DB19832	*virescens*	2	all genes
DB20801	*melisellensis*	1	complete genome
DB22224	*lusitanicus*	2	all genes
DB22633	*guadarramae*	2	all genes
DB24380	*liolepis*	2	all genes
DB25305	*vaucheri*_SL	2	all genes
DB26218	*raffonei*	2	all genes
DB26449	*waglerianus*	1	no ND1, partial ND2
DB27608	OU1_AA	1	complete genome
DB28353	*peloponnesiacus*_E	2	all genes
DB28361	*erhardii*	2	all genes
DB28371	*peloponnesiacus*_W	2	all genes
DB28386	*levendis*	2	all genes
DB28394	*cretensis*	1	complete genome
DB28401	*ionicus*	7	all genes
DB28481	*gaigeae*	1	complete genome
DB28514	*milensis*	1	complete genome
DB5187	*atratus*	2	all genes
DB6975	*vaucheri*_SS	2	all genes
DB7019	*hispanicus*_Gal	2	all genes
DB7063	*hispanicus*_AM	1	complete genome
FH	*filfolensis*	2	all genes
M151	*muralis*_GC	2	all genes
NL24	*muralis*_SA	1	complete genome

PB01	*bocagei*	2	all genes
PF2	*tiliguerta*_S	2	all genes
TRC3	*siculus*_S	2	all genes

* ‘all genes’ means that all genes were present, but the control region was lacking and the mitochondrial genome was therefore not complete.

Supplementary Table 4 | Summary of all introgression events among *Podarcis* species (see Fig. 3b, and Supplementary Fig. 12).

#	Testing topology (W, X), Y), O;	D-statistics (z-score)	QuIBL (BIC)	phyloNet*	qpGraph**	admix. graph (min. error)***	shown in Figure
1	(WestIslands, Balkan), MurIber), O;	0.062 (20.61)	36.71	Yes	Yes	-12945.29	Supplementary Fig. 14
2	(Iberian, *muralis*), Balearic), O;	0.360 (58.53)	19.45	Yes	Yes	-12945.29	Supplementary Fig. 14
3	(*siculus*, *muralis*), Balkan), O;	0.054 (20.71)	114.39	Yes	Yes	-3385.31	Fig. 4
4	(Sicilian, *filfolensis*), *muralis*), O;	0.242 (30.49)	36.59	Yes	Yes	-3385.31	Fig. 4
5	(Sicilian, *filfolensis*), *siculus*), O;	0.163 (22.73)	23.55	Yes	Yes	-3385.31	Fig. 4
6	(SicMalt, Balkan), WestIslands), O;	0.102 (28.98)	48.78	Yes	Yes	-795.3	Supplementary Fig. 15
7	(*milensis*, *gaigeae*), *erhardii*), O;	0.041 (10.75)	0.97	Yes	Yes	-2462.13	Supplementary Fig. 16
8	(*gaigeae*, *milensis*), *tauricus*), O;	0.083 (26.48)	118.07	Yes	Yes	-2462.13	Supplementary Fig. 16
9	(*pelop*_W, *pelop*_E), *cretensis*), O;	0.224 (65.95)	183.48	Yes	Yes	-12372.77	Supplementary Fig. 17
	(*pelop*_W, *pelop*_E), *levendis*), O;	0.305 (74.09)	331.67	Yes	Yes	-12372.77	Supplementary Fig. 17
10	(*lusitanicus*, *bocagei*), *guadarramae*), O;	0.163 (25.47)	8.70	Yes	Yes	-846.81	Supplementary Fig. 18
	(*guadarramae*, *lusitanicus*), *virescens*), O;	0.050 (10.82)	41.89	Yes	Yes	-846.81	Supplementary Fig. 18
11	(*vaucheri*, *hispanicus*), BocClade), O;	0.107 (24.14)	23.24	Yes	Yes	-2942.29	Supplementary Fig. 19
12	(*vaucheri*_SL, *vaucheri*_SS), BocClade), O;	0.066 (24.80)	16.45	Yes	Yes	-2942.29	Supplementary Fig. 19
13	(*virescens*, BocLus), *carbonelli*), O;	0.086 (14.30)	32.08	Yes	Yes	-4323.67	Supplementary Fig. 20
14	(*carbonelli*, BocClade), *atratus*), O;	0.213 (39.18)	31.22	Yes	Yes	-4323.67	Supplementary Fig. 20
	(*carbonelli*, HisClade), *atratus*), O;	0.220 (39.92)	37.12	Yes	Yes	-4323.67	Supplementary Fig. 21
15	(*muralis*_SP, *muralis*_GC), Iberian), O;	0.068 (9.81)	6.11	Yes	Yes	-184.35	Supplementary Fig. 21

*support of the introgression event by the phyloNet method; ‘Yes’ signifies support;

**support of the introgression event by the qpGraph method; ‘Yes’ signifies support;

***minimal errors between best-fitting scenario and the topology of the consensus tree; Note that multiple testing topologies can be comprised in a single minimal error calculation;

Abbreviations: Balearic, Balearic subclade; Balkan, Balkan group; BocClade, *P. bocagei* clade (includes *P. bocagei*, *P. guadarramae*, *P. lusitanicus*, and *P. virescens*); BocLus, *P. bocagei* and *P. lusitanicus*; HisClade, *P. hispanicus* and *P. vaucheri*; Iberian, Iberian group; MurIber, *P. muralis* and species of the Iberian group; O, outgroup; *pelop*, *P. peloponnesiacus*; Sicilian, Sicilian subclade; SicMalt, Sicilian-Maltese group; WestIslands, Western Islands group;

Supplementary Table 5 | Distribution of average dN/dS ratios for *Podarcis* lineages of genes derived from an introgression event (foreground) and genes with a history consistent with the consensus phylogeny (background).

Taxa	dN/dS (Foreground)	dN/dS (Background)	Significance level
Western Islands group	0.2872	0.2713	*P* = 0.105
Sicilian subclade	0.2576	0.2539	*P* = 0.352
Iberian group	**0.2737**	**0.2514**	***P* = 0.018***
P. siculus	0.2744	0.3037	*P* = 0.177
P. muralis SP	**0.3203**	**0.2430**	***P* < 0.010***
P. milensis	**0.3249**	**0.2555**	***P* = 0.006***
P. tauricus	0.2927	0.2765	*P* = 0.095
P. peloponnesiacus E	0.2785	0.2694	*P* = 0.083
P. carbonelli	0.2937	0.2902	*P* = 0.422
P. guadarramae	0.2917	0.3017	*P* = 0.270
P. vaucheri	0.2932	0.2856	*P* = 0.314
P. vaucheri SL	**0.2960**	**0.2719**	***P* = 0.028***

The average dS for foreground and background genes are 0.0263 and 0.0269, respectively. *P* values indicate significance levels derived from 1,000 iterations of permutation tests. Significant comparisons (P < 0.05) with dN/dS values higher in foreground genes are highlighted in bold. Supplementary Fig. 6 shows the distribution of the dN/dS values.

Supplementary Table 6 | Gene list in the genomic block (candidate region) on Chromosome 15.

Ensembl ID	Gene symbol
POMURG00000022601	-
POMURG00000022602	*PLPBP*
POMURG00000022603	-
POMURG00000022604	*ERLIN2*
POMURG00000022605	-
POMURG00000022606	*ZNF703*
POMURG00000022607	-
POMURG00000022608	*FCN1-A*
POMURG00000022609	-
POMURG00000022610	-
POMURG00000022611	*KCNU1*
POMURG00000022612	*KCNU1_3*
POMURG00000022613	*KCNU1_2*
POMURG00000022614	-
POMURG00000022615	-
POMURG00000022616	*UNC5D*
POMURG00000022617	*UNC5D_2*
POMURG00000022618	*TRY2_2*
POMURG00000022619	*SMCHD1*
POMURG00000022620	-
POMURG00000022621	*DUSP26*
POMURG00000022622	*RNF122*
POMURG00000022623	*TTI2*
POMURG00000022624	*MAK16*
POMURG00000022625	-
POMURG00000022626	*MRC1_7*
POMURG00000022627	*ATP6V1B*
POMURG00000022628	*LZTS1*
POMURG00000022629	-
POMURG00000022630	*IL36RN*
POMURG00000022631	*IL36RNIL1RN*
POMURG00000022632	*BRF2_2*
POMURG00000022633	-
POMURG00000022634	*BRF2_3*
POMURG00000022635	*BRF2*
POMURG00000022636	*RAB11*
POMURG00000022637	-
POMURG00000022638	*GOT1L1*
POMURG00000022639	*GOT1_2*

Supplementary Table 7 | Permits for specimen collection.

Country / Region	Permit ID	Issuing authority
Greece	166660/299	Ministry of Environment and Energy
Italy	DPN-2009-0005106, PNM-2012-0009747 and PNM-2017- 0008287	Ministry of Environment
Corsica	2A-2019-04-08-002 and 2B-2019-04-08-011	Direction régionale de l'environnement, de l'aménagement et du logement (DREAL) de Corse
Portugal	66/2008/CAPT, 171/2012/CAPT, and 498/2015/CAPT	Instituto para a Consrevação da Natureza (ICN)
Spain	OAEN/SVSIA/avp_10_169_aut	Organismo Autónomo Espacios Naturales de Castilla – La Mancha
Spain	EP/SA/373/2016	Delegación Territorial de Salamanca, Junta de Castilla y León
Spain	500201/24/2014/3218	Departamento de Agricultura, Ganadería y Medio Ambiente, Gobierno de Aragón
Spain	AUF20190018	Consejería de Empleo, Universidades, Empresa y Medio Ambiente, Región de Murcia
Spain	CAP 54/2011 and 737/2011	Conselleria de Medi Ambienti Mobilitat, Govern de les Illes Balears
Spain	SGB/FOA/AFR	Consejería de Medio Ambiente, Junta de Andalucía
Morocco	10/2014/HCEFLCD/DLCDPN/DPRN/CFF	Le Haut Commissaire aux Eaux et Forêts et à la Lutte Contre la Désertification du Royaume de Maroc
Slovenia	35601-66/2013-4	Ministry of Agriculture and the Environment

Supplementary information references

1. Kaliontzopoulou, A., Pinho, C., Harris, D. J. & Carretero, M. A. When cryptic diversity blurs the picture: a cautionary tale from Iberian and North African Podarcis wall lizards. *Biol J Linn Soc* **103**, 779-800 (2011).
2. Bassitta, M. *et al.* Multilocus and morphological analysis of south-eastern Iberian Wall lizards (Squamata, Podarcis). *Zool. Scr.* **n/a** (2020).
3. Salvi, D., Harris, D. J., Kaliontzopoulou, A., Carretero, M. A. & Pinho, C. Persistence across Pleistocene ice ages in Mediterranean and extra-Mediterranean refugia: phylogeographic insights from the common wall lizard. *BMC Evol Biol* **13**, 147 (2013).
4. Spilani, L. *et al.* Multigene phylogeny, phylogeography and population structure of Podarcis cretensis species group in south Balkans. *Mol Phylogenet Evol* **138**, 193-204 (2019).
5. Podnar, M., Mayer, W. & Tvrtkovic, N. Phylogeography of the Italian wall lizard, Podarcis sicula, as revealed by mitochondrial DNA sequences. *Mol Ecol* **14**, 575-588 (2005).
6. Salvi, D., Pinho, C. & Harris, D. J. Digging up the roots of an insular hotspot of genetic diversity: decoupled mito-nuclear histories in the evolution of the Corsican-Sardinian endemic lizard Podarcis tiliguerta. *BMC Evol Biol* **17**, 63 (2017).
7. Caeiro-Dias, G. *et al.* Lack of congruence of genetic and niche divergence in Podarcis hispanicus complex. *J. Zool. Syst. Evol. Res.* **56**, 479-492 (2018).
