# Supplementary material for: Extensive introgression and mosaic genomes of Mediterranean endemic lizards
Source: Nat Commun. 2021 May 12;12:2762. doi: 10.1038/s41467-021-22949-9 (PMC8114931; doi:10.1038/s41467-021-22949-9)
Supplement: Supplementary file 3 — Reporting Summary [file 41467_2021_22949_MOESM3_ESM.pdf]

nature research

Corresponding author(s): Tobias Uller, Weizhao Yang

Last updated by author(s): Mar 17, 2021

Reporting Summary

Nature Research wishes to improve the reproducibility of the work that we publish. This form provides structure for consistency and transparency in reporting. For further information on Nature Research policies, see our Editorial Policies and the Editorial Policy Checklist.

Statistics

For all statistical analyses, confirm that the following items are present in the figure legend, table legend, main text, or Methods section.

n/a	Confirmed	
☐	☒	The exact sample size (*n*) for each experimental group/condition, given as a discrete number and unit of measurement
☐	☒	A statement on whether measurements were taken from distinct samples or whether the same sample was measured repeatedly
☐	☒	The statistical test(s) used AND whether they are one- or two-sided<br>*Only common tests should be described solely by name; describe more complex techniques in the Methods section.*
☒	☐	A description of all covariates tested
☐	☒	A description of any assumptions or corrections, such as tests of normality and adjustment for multiple comparisons
☐	☒	A full description of the statistical parameters including central tendency (e.g. means) or other basic estimates (e.g. regression coefficient) AND variation (e.g. standard deviation) or associated estimates of uncertainty (e.g. confidence intervals)
☐	☒	For null hypothesis testing, the test statistic (e.g. *F*, *t*, *r*) with confidence intervals, effect sizes, degrees of freedom and *P* value noted<br>*Give P values as exact values whenever suitable.*
☐	☒	For Bayesian analysis, information on the choice of priors and Markov chain Monte Carlo settings
☒	☐	For hierarchical and complex designs, identification of the appropriate level for tests and full reporting of outcomes
☒	☐	Estimates of effect sizes (e.g. Cohen's *d*, Pearson's *r*), indicating how they were calculated

Our web collection on statistics for biologists contains articles on many of the points above.

Software and code

Policy information about availability of computer code

Data collection	Raw sequence data were obtained by Illumina Sequencing.
Data analysis	All data were analysed using publicly available software and statistical packages. No new software or custom code has been developed during the course of this study. The sofware used in this study is the following: AdmixTools v6.0; ASTRAL-III v5.7.1; BEAST2 v2.6.2; BEAGLE V4.1; bwa-mem v0.7.1; FastQC v0.11.8; fineSTRUCTURE v4; GATK v3.8; genomics_general v0.3; IQTree v1.6.12; MUSCLE v3.8.425; NOVOPlasty v4.2; PAML v4.9j; phyloNet v3.8.2; PSMC v0.6.5; QuIBL; R v3.63; R package ape v5.3; R package Admixgraph v1.0.2; R package treePar v3.3; RevBayes v1.1.0; r8s v1.70; trimmomatic v0.38; Twisst v0.1.

For manuscripts utilizing custom algorithms or software that are central to the research but not yet described in published literature, software must be made available to editors and reviewers. We strongly encourage code deposition in a community repository (e.g. GitHub). See the Nature Research guidelines for submitting code & software for further information.

Data

Policy information about availability of data

All manuscripts must include a data availability statement. This statement should provide the following information, where applicable:

- Accession codes, unique identifiers, or web links for publicly available datasets
- A list of figures that have associated raw data
- A description of any restrictions on data availability

All sequence data generated in this study have been deposited in NCBI Short Reads Archive (SRA) with accession number PRJNA715201.

Field-specific reporting

Please select the one below that is the best fit for your research. If you are not sure, read the appropriate sections before making your selection.

☐ Life sciences ☐ Behavioural & social sciences ☒ Ecological, evolutionary & environmental sciences

For a reference copy of the document with all sections, see nature.com/documents/nr-reporting-summary-flat.pdf

Ecological, evolutionary & environmental sciences study design

All studies must disclose on these points even when the disclosure is negative.

Study description	We conducted analysis of whole genome sequences of 34 lineages of wall lizards to reconstruct evolutionary history, identified reticulation events, and quantified patterns of genomic introgression.
Research sample	Whole genome sequences of 34 major lineages (including all 26 recognized species) of wall lizard and two additional species as outgroup. We selected one representative individual per lineage/species, collected a tissue sample and extracted DNA. All selected individuals were fully-grown, adult males.
Sampling strategy	We sampled all major lineages of this clade, covering the extant diversity within the genus Podarcis
Data collection	Samples were collected by the authors under valid licenses (see Supplementary Material for a list of sampling permits). Tissue samples were collected in the field by taking biopsies of tail tips (ca. 1 cm) and storing it in ethanol. From these tissue samples, we extracted DNA using standard methods (see Methods section) and subjected it to next-generation sequencing.
Timing and spatial scale	Tissue samples were collected in the field between 2008-2019. Sampling occurred across the entire range of the clade, i.e., the Mediterranean Basin including Spain, Portugal, Morocco, Italy, Corsica, Greece and Slovenia. The sampling was stopped when all major lineages were covered with one sample.
Data exclusions	No samples/data were excluded
Reproducibility	The study is not experimental. Reproducibility does not apply to this study as the experimental design precludes repeated measurements. However, by making all data and methodology publicly available, other researchers can reproduce the results reported in this study.
Randomization	The study is not experimental. Randomization does not apply to this study since it does not follow a case-control design.
Blinding	The generation of DNA sequence data is blind with respect to the analyst. All analyses followed standard procedures in the field and blinding is not common practice in the analyses of phylogenomic data.

Did the study involve field work? ☒ Yes ☐ No

Field work, collection and transport

Field conditions	Field work was conducted during spring/summer under conditions suitable for lizard activity. In the context of this study of genomic DNA, environmental conditions are not relevant.
Location	Field work took place throughout the Mediterranean Basin. Exact locations are provided in Supplementary Table 1.
Access & import/export	All samples were collected and imported under local and EU permits (Supplementary Table 5)
Disturbance	The field work associated with this study did not cause any disturbance.

Reporting for specific materials, systems and methods

We require information from authors about some types of materials, experimental systems and methods used in many studies. Here, indicate whether each material, system or method listed is relevant to your study. If you are not sure if a list item applies to your research, read the appropriate section before selecting a response.

Materials & experimental systems

n/a	Involved in the study
☒	☐ Antibodies
☒	☐ Eukaryotic cell lines
☒	☐ Palaeontology and archaeology
☐	☒ Animals and other organisms
☒	☐ Human research participants
☒	☐ Clinical data
☒	☐ Dual use research of concern

Methods

n/a	Involved in the study
☒	☐ ChIP-seq
☒	☐ Flow cytometry
☒	☐ MRI-based neuroimaging

Animals and other organisms

Policy information about studies involving animals; ARRIVE guidelines recommended for reporting animal research

Laboratory animals	No laboratory animals were used in this study.
Wild animals	Wild, adult, male lizards were captured by noosing. Tissue samples were collected directly in the field by removing approximately 1 cm of the tail. Upon tissue collections, lizards were immediately released again at the site of capture. All procedures were conducted under local (e.g., national/regional) and EU ethical licenses.
Field-collected samples	No live animals were removed from the field.
Ethics oversight	All the work in this study was conducted under ethical permits approved under national and EU legislation. See Supplementary Table 7 for a complete list.

Note that full information on the approval of the study protocol must also be provided in the manuscript.
